# Toxoplasmosis in the Era of Targeted Immunotherapy: A Systematic Review of Emerging Cases Linked to Biologics and Small Molecules in Autoimmune Diseases, Oncology and Transplantation

**DOI:** 10.3390/pathogens14101001

**Published:** 2025-10-03

**Authors:** Stephanie M. Cho, Jose G. Montoya, Despina G. Contopoulos-Ioannidis

**Affiliations:** 1 College of Osteopathic Medicine of the Pacific, Western University of Health Sciences, Pomona, CA 91766, USA; stephanie.cho@westernu.edu; 2 Dr. Jack S. Remington Laboratory for Specialty Diagnostics, Sutter Health, Palo Alto Medical Foundation, Palo Alto, CA 94301, USA; jose.montoya2@sutterhealth.org

**Keywords:** toxoplasmosis, *Toxoplasma*, *T. gondii*, targeted immunotherapy, biologics, CAR T-cell, small molecules, systematic review

## Abstract

A systematic review of toxoplasmosis cases in patients receiving targeted immunotherapy with biologics or small molecules was performed. This systematic review searched for case reports, case series and observational studies in PubMed; last search was on 19 July 2025. The review identified 46 toxoplasmosis cases among patients receiving biologics (including CAR T-Cell Therapies) or small molecules for diverse autoimmune, oncologic and transplant conditions. These cases were reported from 18 countries, including the United States and several European countries. Most patients developed severe disease. Fifty percent (23/46) presented with cerebral toxoplasmosis, 33% (15/46) with ocular toxoplasmosis, 7% (3/46) with lymphadenopathy, 4% (2/46) with disseminated disease, 2% (1/46) with both cerebral and ocular disease, 2% (1/46) with pneumonic toxoplasmosis, and 2% (1/46) with severe fetal congenital toxoplasmosis. Among those were also four cases with fatal outcomes due to toxoplasmosis and eight cases with permanent ocular or neurological deficits. In addition, there was a case of fetal congenital toxoplasmosis that occurred despite maternal discontinuation of adalimumab five months before conception, resulting in elective pregnancy termination due to severe fetal cerebral disease. Overall, 44% (20/46) of cases were due to reactivation of chronic latent *Toxoplasma* infections and 39% (18/46) due to acute primary infections; 17% did not report this information. One case of disseminated acute toxoplasmosis was also identified after eating wild boar sausages, and two cases of severe acute ocular toxoplasmosis after eating undercooked venison meat, and undercooked unspecified type of meat respectively, while on small molecules or biologics. Details on the clinical presentations, management and clinical outcomes of these cases were reported. Recommendations for the management of toxoplasmosis in patients with targeted immunotherapies were also provided. Health care providers should consider toxoplasmosis in patients on biologics or small molecules who present with compatible clinical syndromes. Prompt diagnosis and treatment can be lifesaving.

## 1. Introduction

*Toxoplasma gondii* is a ubiquitous protozoan parasite with a worldwide distribution [1,2,3,4]. The organism is commonly transmitted to humans via the oral route. One common route of transmission includes the accidental ingestion of cat excreted oocysts in soil via eating unwashed fruits or vegetables, drinking untreated water or during gardening. Transmission can also occur through the ingestion of tissue cysts from eating undercooked meat. *T. gondii* is estimated to infect almost a third of the world’s population [5]. 

Primary infections in immunocompetent individuals are often either asymptomatic or can present with a mononucleosis-like illness. However, even immunocompetent patients can develop serious disease from toxoplasmosis [2,3,4,6]. Immunocompromised patients have a higher risk for severe and life-threatening toxoplasmosis, after acute primary infections or from reactivation of latent infections [7,8,9]. The clinical spectrum of toxoplasmosis is wide, including encephalitis, pneumonia, ocular disease, myopericarditis, disseminated disease, hepatitis, myositis, tick-borne-disease like illness (with leukopenia, lymphopenia, thrombocytopenia, and transaminitis), or congenital infections [2]. 

*Toxoplasma gondii* is a globally prevalent parasite whose clinical manifestations are both preventable and treatable [10]. With the increasing use of biologics and small molecules for autoimmune, oncologic, or transplant-related conditions, there is growing need to understand the risk of toxoplasmosis associated with the use of these treatments [11,12,13]. We conducted a systematic review of toxoplasmosis cases in patients receiving biologics (including CAR T-Cell Therapies) or small molecules for autoimmune, oncologic or transplant-related conditions, to better characterize this risk and provide recommendations for prevention and management.

## 2. Methods

### 2.1. Search Strategy

In this systematic review PubMed was searched using two search strategies (search #1 and search #2) shown in Table S1. Last PubMed search was on 19 July 2025. The review targeted toxoplasmosis cases across diverse classes of biologics or small molecules targeted immunotherapies, using both the drug class names and specific drug names in these classes. The list of the 106 individual biologic agents or small molecules searched is shown in Table S2. The reference lists of the included articles were also screened. We followed the PRISMA guidelines of reporting. The PRISMA checklist is shown in Table S3. This systematic review is registered in the Open Science Framework (OSF) Registry of the Center for Open Science (https://osf.io/jz2fy/; accessed on 18 September 2025).

### 2.2. Inclusion and Exclusion Criteria

This systematic review considered case reports, case series or observational studies, where the targeted immunotherapies with biologics or small molecules were administered for autoimmune, oncologic or transplant-related conditions. Only cases where the targeted immunotherapies were started prior to the onset of toxoplasmosis were considered. Both English and non-English languages were included; non-English papers were analyzed using automated translation tools. Review articles and editorials were excluded. 

### 2.3. Data Extraction

The following information was extracted from the eligible articles: patient’s age, country (case), sex, underlying medical condition, age, targeted immunotherapy used, targeted immunotherapy drug class, duration of targeted immunotherapy prior to toxoplasmosis onset, toxoplasmosis chief complaint at presentation, toxoplasmosis clinical manifestations and pertinent laboratory findings, diagnostic confirmation of toxoplasmosis diagnosis, modification of targeted immunotherapy during active toxoplasmosis, anti-*Toxoplasma* treatment (medications and duration thereof), toxoplasmosis clinical outcome, and secondary anti-*Toxoplasma* prophylaxis (after completion of anti-*Toxoplasma* therapy). 

Data were extracted independently by S.M.C and D.G.C.-I.; the final adjudication of eligible cases and extracted data was carried out according to consensus between SC, DCI and JGM. The PRISMA guidelines of reporting for systematic reviews were followed. Results were analyzed using descriptive statistics. A compilation table of short vignettes was created to highlight pertinent clinical presentations, course, diagnosis, management, and outcomes of those toxoplasmosis cases. Recommendations for the diagnosis and management of toxoplasmosis were also provided. 

## 3. Results

**Figure 1:**
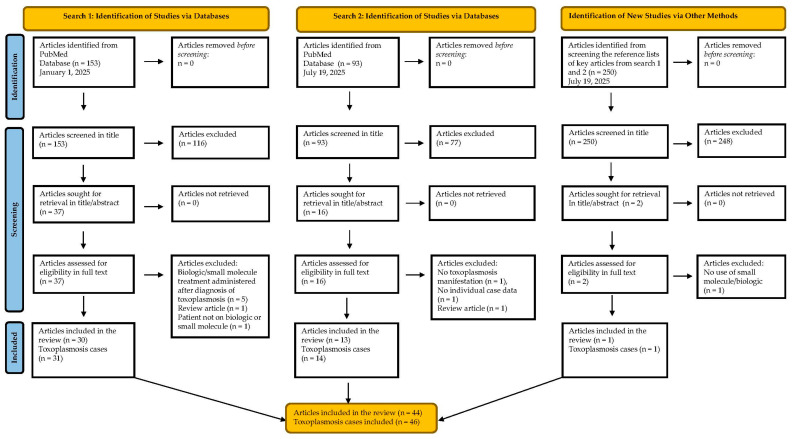
Flow diagram of the literature search for the included studies (PRISMA flowchart).

Two collective search strategies (search #1 and search #2) retrieved 246 articles in addition to two articles that were identified through the reference list search of key articles, which were screened at the title level. Among those, 55 potentially eligible articles were further screened at full text level, and 44 papers were considered eligible for inclusion (Figure 1) pertaining to 46 patients who developed toxoplasmosis while receiving targeted immunotherapies, or shortly after. A reference list of all included papers is shown in Table S4.

### 3.1. Characteristics of Toxoplasmosis Cases

The toxoplasmosis cases were reported from 18 countries, including Australia, Belgium, Brazil, Colombia, France, Germany, India, Ireland, Italy, Netherlands, Portugal, Slovenia, Spain, Switzerland, Turkey, United Kingdom, United States and Venezuela. Approximately 52% of the patients were female (n = 24) and of the cases with reported age of diagnosis, the age range was 17 to 86 years, with median age 52 years (Table 1).

### 3.2. Underlying Autoimmune and Non-Autoimmune Conditions

Biologics or small molecules were administered for the following six disease categories: rheumatological conditions (n = 16, including cases with rheumatoid arthritis, ankylosing spondylitis, dermatomyositis, juvenile idiopathic arthritis), oncologic conditions (n = 12, including cases with chronic leukemia, lymphoma, polycythemia vera, lung cancer), transplant-related conditions (n = 5, including hematopoietic stem cell transplant (HSCT), kidney transplant, liver transplant, graft versus host disease post HSCT), dermatological autoimmune conditions (n = 5, including cases with pemphigus vulgaris, psoriasis, necrotizing vasculitis), neurologic autoimmune conditions (n = 3, multiple sclerosis), gastrointestinal autoimmune conditions (n = 3, including cases with Crohn’s Disease, ulcerative colitis), pulmonary autoimmune conditions (n=1, case with sarcoidosis), and combination of rheumatologic and oncologic conditions (n = 1) (Table 1).

### 3.3. Targeted Immunotherapies (Biologic Agents or Small Molecules)

This systematic review identified 38 toxoplasmosis cases associated with biologic agents and eight cases associated with small molecules. The identified biologics or small molecules targeted immunotherapies included abatacept (n = 1), adalimumab (n = 11), alemtuzumab (n = 4), CAR T-Cells (n = 3), belatacept (n = 2), erlotinib (n = 1), etanercept (n = 1), fingolimod (n = 1), golimumab (n = 1), imatinib (n = 1), infliximab (n = 4), iscalimab (n = 1), ixekizumab (n = 1), JAK3 inhibitors not specified (n = 1), natalizumab (n = 1), rituximab (n = 5), ruxolitinib (n = 3), trametinib (n=1), TNF-a inhibitor not specified (n = 1), and ustekinumab (n = 2).

Identified toxoplasmosis cases were associated with the following classes of biologic agents: (a) tumor necrosis factor-a inhibitors (anti-TNF-a) (n = 18), (b) T-cell co-stimulation inhibitors (n = 3), (c) anti-CD20 (n = 5), (d) anti-CD52 (n = 4), (e) CAR-T-cell therapies (n = 3), (f) IL-12/IL-23 inhibitor (n = 2); (g) interleukin-17 (IL-17-a) inhibitor (n = 1), (h) anti-CD40 (n = 1), and (i) cell adhesion molecule inhibitors/integrin receptor inhibitors (n = 1). 

Moreover, identified toxoplasmosis cases were also associated with the following classes of small molecules: (a) janus kinase (JAK) inhibitors (n = 4), (b) mitogen-activated extracellular signal regulated kinase (MEK) inhibitors (n = 1), (c) tyrosine kinase inhibitors (n = 2), (d) sphingosine 1-phosphate receptor modulator (n = 1) (Table 1).

Some patients had been on more than one targeted immunotherapy in the recent period prior to the onset of toxoplasmosis. No toxoplasmosis cases were identified with checkpoint inhibitor biologics in this review.

### 3.4. Duration of Treatment with the Targeted Immunotherapies Prior to Toxoplasmosis Onset

The duration of targeted immunotherapy treatment prior to the onset of toxoplasmosis varied according to whether the toxoplasmosis was due to reactivation of a chronic latent infection or due to an acute primary infection. In toxoplasmosis cases due to reactivation of chronic latent *Toxoplasma* infection, the median reported duration of the targeted immunotherapy treatment was four months (range 1–12 months). Reactivations were noted to occur after recent introduction of the targeted immunotherapies to other immunosuppressive agents or after recent increase in their dose. In cases of toxoplasmosis after an acute primary *Toxoplasma* infection, the median duration was 9 months, and the reported duration varied more (range 0.5–48 months) (Table 1).

### 3.5. Attribution of Toxoplasmosis to the Targeted Immunotherapy

In 54% (25/46) of cases, toxoplasmosis was clearly attributed to the targeted immunotherapy as patients were/had receiving either monotherapy of the targeted immunotherapy (n = 20); or toxoplasmosis developed shortly after the recent addition of the targeted immunotherapy (n = 4) or recent increase in their dose (n = 1). 

In the remaining 46% (21/46) of cases, toxoplasmosis was likely at least co-attributed to the targeted immunotherapy, as patients were receiving targeted immunotherapy in addition to other immunosuppressive agents at the time of toxoplasmosis diagnosis (Table 1). 

### 3.6. Reactivation of Latent Infections vs. Acute Primary Toxoplasma Infections

In 44% (20/46) of cases, toxoplasmosis occurred in the setting of reactivation of preexisting chronic latent *Toxoplasma* infections, while in 39% (18/46), in the setting of acute primary *Toxoplasma* infections. In the remaining 17% (8/46) of cases, there was not enough information provided for the determination (Table 1).

### 3.7. Acute Toxoplasmosis Cases After Consumption of Undercooked Meat, Including Wild Game Meat

Three severe acute toxoplasmosis cases occurred after high-risk *T. gondii* exposures through food ingestion of undercooked meat. One case occurred in the US in a patient with metastatic lung cancer on erlotinib monotherapy, who ate undercooked wild venison and developed severe bilateral ocular toxoplasmosis [37]. Another case in the US occurred in a patient on trametinib (among other recent immunosuppressants) who ate wild boar sausages and developed disseminated toxoplasmosis (with cerebral disease with multiple ring-enhancing brain lesions, severe retinitis with vision loss, myocarditis and myositis) [6]. A third case, in France, occurred in a kidney transplant patient on belatacept (among other immunosuppressants) who had been on biologics for 11 years and after consumption of undercooked meat developed severe ocular toxoplasmosis [40]. 

### 3.8. Clinical Syndromes

Overall, 50% (23/46) of reported cases presented with cerebral toxoplasmosis, 33% (15/46) with ocular toxoplasmosis, 7% (3/46) with lymphadenopathy [14,44,45], 2 cases (4%) with disseminated disease (including one case of disseminated disease with severe lung involvement and respiratory failure) [6,33], one case (2%) with pneumonic toxoplasmosis [32], one case (2%) with both cerebral and ocular disease [42], and one case (2%) led to fetal congenital toxoplasmosis with elective pregnancy termination due to severe central nervous system (CNS) disease [10]. Some cases had more than one organ involved (Table 1).

The toxoplasmosis clinical presentations, according to the biologic agents or small molecules, are shown in Table 2. Serious clinical manifestations were reported with almost all classes of reported biologics or small molecules.

### 3.9. Clinical and Radiologic Manifestations

The neurologic manifestations of cerebral toxoplasmosis cases included encephalitis; altered mental status; cognitive impairment (including confusion, memory loss) [8,23,27,29,31,34,38,46]; acute psychosis (including manic episodes [16]); focal neurologic findings (including hemiparesis/hemiplegia [8,17,21,23,25,29,31,39,41,42,51]; paraparesis [15]; cranial nerve palsies (with dysarthria, dysphagia, facial palsy, homonymous hemianopsia) [16,21,23,25,29,46]; gait incoordination [6,8,15,29,35,42]; aphasia [23]; apraxia [27]; fine motor difficulties [6,15]; headache [15,16,25,29,32,33,43,53,54]; and seizures [25,26,35,54]. Reported manifestations of pneumonic toxoplasmosis cases also included respiratory failure. Reported manifestations of ocular toxoplasmosis were bilateral or unilateral (with floaters, blurred vision, scotomas, and decreased visual acuity). Some patients developed permanent vision loss [18,19,20,24,40,47,50] or permanent neurocognitive deficits [46] despite anti-*Toxoplasma* treatment. Cardiac manifestations in the setting of toxoplasmic myocarditis included dyspnea and orthopnea [6]. Reported general manifestations included muscle pains (in the setting of toxoplasmic myositis [6]) and lymphadenopathy [14,44,45]. Constitutional symptoms included mononucleosis like symptoms with fever, malaise, headache, and chills. Laboratory abnormalities included (among other) cerebrospinal fluid (CSF) lymphocytic pleocytosis, elevated CSF protein, and/or peripheral lymphopenia [8]. There was also a case of severe fetal congenital toxoplasmosis with disseminated disease [10] (Tables S5 and S6).

The reported brain imaging abnormalities in cases of cerebral toxoplasmosis included multiple supratentorial lesions (bilateral or unilateral) [16], large space-occupying lesions (with brain abscess- or brain tumor-like lesions) with or without ring enhancement [8], hypodense white matter lesions (in thalamic and parieto-occipital areas) [16], and heterogeneous-appearing lesions on T2/FLAIR images. The reported lung imaging findings of pulmonic toxoplasmosis included bilateral interstitial lung infiltrates and/or diffuse ground glass opacities in the chest CT, which mimicked atypical pneumonias or *Pneumocystis jirovecii* pneumonia (Tables S5 and S6).

### 3.10. Diagnostic Confirmation 

In 61% (28/46) of the cases, multimodal diagnostic evaluations were used, with different combinations of serology, polymerase chain reaction (PCR), next generation sequencing, and/or histopathology/immunohistochemistry. (Table 3) The diagnosis of active toxoplasmosis was confirmed by serological testing (*T. gondii* antibodies, with or without IgG avidity) in 72% (33/46) of cases, by PCR (*T. gondii* DNA PCR in CSF, blood, ocular fluid or amniotic fluid) in 46% (21/46) of cases, by next generation sequencing in 2% (1/46) [43] of cases, or by histopathology of tissue biopsies (with *T. gondii* immunohistochemistry, or documentation of ruptured bradyzoite tissue cysts or tachyzoites) in 33% (15/46) of cases, including post-mortem biopsies. 

Serologic confirmation of reactivation of chronic latent *T. gondii* infection was based on documentation of positive *T. gondii* IgG, often at high titers, with negative IgM. Serologic confirmation of acute primary *T. gondii* infection was based on one of the following: (a) documentation at presentation of positive *T. gondii* IgG and IgM (and/or positive *T. gondii* IgA and/or low *T. gondii* IgG avidity), (b) documentation of seroconversion from *T. gondii* IgG negative status to *T. gondii* IgG positive status [19], (c) documentation of maturation of *T. gondii* IgG avidity from low to high avidity [14,45], or (d) documentation of rising *T. gondii* IgG (and/or IgM) antibody titers. Retrospective testing of serum samples available prior to the clinical presentation was helpful in some cases in establishing the diagnosis of acute toxoplasmosis [10,19].

Next-generation cell-free DNA sequencing (Karius Test, Karius Inc, Redwood City, California, USA [43]) helped to establish the diagnosis of acute toxoplasmosis in one case of cerebral toxoplasmosis with high positive *T. gondii* IgM titers, but negative *T. gondii* IgG and negative *T. gondii* PCR in the patient’s CSF. 

The clinical and radiographic response to anti-*Toxoplasma* therapy further supported the diagnosis of active toxoplasmosis in some cases. Several cases confirmed the diagnosis of cerebral toxoplasmosis with brain biopsy, even when *T. gondii* serology was negative in the context of profound lymphopenia [34,53]. Moreover, the review found cases of cerebral toxoplasmosis where the CSF analysis was normal and/or the CSF *T. gondii* PCR was negative, despite biopsy-confirmed [26,42] or autopsy-confirmed cerebral toxoplasmosis [31].

The case of fetal congenital toxoplasmosis was confirmed by positive *T. gondii* PCR of the amniotic fluid [10]. There were multiple brain abnormalities in the fetal ultrasound (right ventriculomegaly and multiple hyperechoic lesions in the cerebral parenchyma) and the fetal MRI (multiple subependymal T2 intense lesions and multiple cortical and subcortical cysts). Also, autopsy confirmed disseminated disease with necrotizing encephalitis with large necrotic and inflammatory lesions in the brain, hepatosplenomegaly and calcifications in adrenal glands. 

### 3.11. Modification of Targeted Immunotherapy at Presentation

After diagnosis of toxoplasmosis, 54% (25/46) of the cases discontinued targeted immunotherapy, either temporarily or permanently. In one cerebral toxoplasmosis case (2%), the dose of the small molecule (ruxolitinib) was continued at a decreased dose [38]. However, this patient subsequently developed Guillain–Barre Syndrome, a complication possibly co-attributed to toxoplasmosis disease. In another two cases (4%) (a case of toxoplasmic lymphadenopathy [44] and a case of ocular toxoplasmosis [30]), the biologic agent was continued throughout the course of toxoplasmosis, with a good outcome. In 17% (8/46) of cases, this information was not reported, and in 22% (10/46) of cases, it was not applicable as the patient had either died early in the course [54], had completed the targeted immunotherapy [16,31,33,34,35,46] targeted therapy was already discontinued due to side effects [32], or pregnancy termination was elected [10] (Table 3). Among the 25 cases who discontinued the targeted immunotherapy due to active toxoplasmosis, 24% (6/25) permanently discontinued all biologics/small molecules [8,15,24,29,43,48], 12% (3/25) discontinued both small molecule/biologic and other immunosuppressants [45,50,53], 8% (2/25) reintroduced them later [14,47], and 8% (2/25) switched therapy to another small molecule [22,51]. In the remaining 48% (12/25) of the cases, further details on the management of targeted immunotherapy after the initial modification of the therapy was not reported [17,18,19,20,21,26,28,39,41,42,49,52] (Table 3).

### 3.12. Anti-Toxoplasma Therapy

The combination regimen of pyrimethamine, sulfadiazine, and folinic acid was used in 39% (18/46) of cases. Trimethoprim/sulfamethoxazole (TMP/SMX) was used in 37% (17/46) of cases. In 13% (6/46) of cases, pyrimethamine was co-administered with other medications, e.g., clindamycin, azithromycin, or dapsone. Atovaquone was used as treatment only in one case (2%) of ocular toxoplasmosis [37]. Information about the anti-*Toxoplasma* treatment was not reported in two cases (4%) [27,36], and treatment was not required in one case (2%) as the patient’s toxoplasmic lymphadenopathy improved with just discontinuation of the biologic agent [45]. Anti-*Toxoplasma* treatment was not used in the one case (2%) of elective pregnancy termination [10] (Table 3).

### 3.13. Duration of Anti-Toxoplasma Therapy

Anti-*Toxoplasma* treatment was most often administered for 6–8 weeks, but the reported duration ranged from 2–16 weeks; a two-week treatment was administered only for a case of toxoplasmic lymphadenopathy (Table 3). 

### 3.14. Clinical Outcomes

Over half (59%; [27/46]) of the toxoplasmosis cases had favorable outcomes by the time of the last follow up reporting either a complete recovery (20% [9/46]) or clinical and/or radiologic improvement (39% [18/46]) (Table 3).

Nevertheless, 37% (17/46) of cases reported unfavorable outcomes. Among those, there were four cases with fatal outcomes due to toxoplasmosis [27,31,33,54], one case of elective pregnancy termination due to severe fetal congenital toxoplasmosis [10], eight cases with permanent deficits (seven with permanent visual deficit [18,19,20,24,40,47,50] and one with permanent neurological deficit [46]) and three cases with complications (Guillain–Barre syndrome likely due to toxoplasmosis [38], brain hemorrhage after the diagnostic brain biopsy [17], and acute psychosis [16]). There was one additional case where the patient expired; however, the cause of death was likely not due to toxoplasmosis as the patient died two months after clearance of the vitritis and return of visual acuity to baseline [37]. The remaining 4% (2/46) of cases did not report the patient outcome (Table 3). 

### 3.15. Secondary Anti-Toxoplasma Prophylaxis

Only 11 cases reported information on secondary anti-*Toxoplasma* prophylaxis; these included cases with severe cerebral toxoplasmosis (n = 3) [8,15,29,35,39,43,53], severe pneumonic toxoplasmosis (n = 1) [32], cerebral and ocular toxoplasmosis [42], and severe vision threatening ocular toxoplasmosis (n = 2) [18,20]. Agents that were used for secondary prophylaxis included TMP/SMX, pyrimethamine/sulfadiazine/folinic acid or atovaquone (Table 3).

A compilation of clinical vignettes, by toxoplasmosis clinical syndromes, for the 46 cases is provided in Table S5. All the raw data for the 46 cases are available in Table S6.

## 4. Discussion

Although the primary goal in developing biologics and small molecules has been to achieve targeted immunomodulation with minimal disruption of the overall immune system, their clinical use remains challenged by infectious complications, including opportunistic pathogens such as *Toxoplasma gondii*. Likewise, while CAR T-cells are specifically engineered to enhance tumor cell killing, these regimens also carry a substantial risk of infection. This risk arises from the lymphodepleting chemotherapy (e.g., fludarabine and cyclophosphamide [55]) administered during the conditioning phase, and, in the case of anti-CD19 CAR T-cell therapy, from the concurrent depletion of normal B cells [56].

In this systematic review, we identified 46 toxoplasmosis cases among patients receiving biologics (including CAR T-cell therapies) or small molecules for diverse autoimmune, oncologic or transplant-related conditions, reported from 18 diverse countries, including the US and several European countries. Most of the reported toxoplasmosis cases were severe, including (a) cases of cerebral toxoplasmosis (with multiple brain lesions with ring enhancement, large space occupying lesions mimicking brain abscess or brain tumor), with encephalitis, mental status changes, acute psychosis, severe motor deficits [with hemiparesis, paraparesis], unsteady gait and cranial nerve palsies [with dysphagia, dysarthria]; (b) cases of ocular toxoplasmosis with vision loss; (c) cases with pneumonic toxoplasmosis; (d) cases with disseminated disease; (e) cases with toxoplasmic lymphadenopathy and mononucleosis-like illness; and (f) a case of severe fetal congenital toxoplasmosis. 

In the absence of prospective registries for toxoplasmosis (and other opportunistic infections) for patients on targeted immunotherapies, the true burden of disease for toxoplasmosis in this patient population will remain unknown.

### 4.1. Fatal Toxoplasmosis Cases

Toxoplasmosis in patients on targeted immunotherapies occasionally can be fatal. We identified four cases with fatal outcomes due to toxoplasmosis [27,31,33,54]. 

The first case was a 63-year old patient from Ireland who developed cerebral toxoplasmosis and progressive multifocal leukoencephalopathy (PML) while on rituximab (among other regimens) for eight months prior to presentation for chronic lymphocytic leukemia [27]. PCR analysis of CSF was positive for both *T. gondii* and JC virus and the immunohistochemistry was positive for JC virus. However, the brain biopsy showed white matter replacement with oligodendroglia intranuclear inclusions adjacent to ruptured toxoplasmic cysts, suggestive of cerebral toxoplasmosis. The patient developed progressive ataxia, dysphagia and dementia and died four weeks after the initial diagnosis. It was not reported if the patient received anti-*Toxoplasma* treatment. 

The second case was a 25-year-old patient from India who developed cerebral toxoplasmosis while on golimumab for sarcoidosis [54]. The patient presented with headache, projectile vomiting and visual blurring, had CSF lymphocytosis, elevated CSF protein and multiple ring-enhancing brain lesions with hemorrhagic foci in the brain MRI. Brain Biopsy and immunohistochemistry confirmed the presence of *T. gondii* trophozoites. Patient was started on TMP/SMX but suffered a status epilepticus episode that was refractory to treatment and died from irreversible anoxic damage.

The third case was a 54-year-old patient from the US, who after CAR T-cell therapy (in addition to other immunosuppressants) for relapsed/refractory diffuse large B cell lymphoma, developed several complications, including hemophagocytic lymphohistiocytosis and multiorgan failure [33]. Patient died on day 70 after CAR T-cell therapy despite aggressive treatment. Autopsy showed disseminated toxoplasmosis with *Toxoplasma* tachyzoites and tissue cysts in the CNS (cerebrum, cerebellum, medulla, cervical spine) and extensive lung involvement (with neutrophilic infiltrates, hemorrhages and *Toxoplasma* cysts and debris and positive *T. gondii* immunohistochemistry). 

The fourth case was a 22-year-old male from South America who developed severe progressive cerebral toxoplasmosis after alemtuzumab based HSCT for AML [31]. Patient died approximately one month post HSCT. Postmortem brain biopsy confirmed the diagnosis of cerebral toxoplasmosis. 

We also identified a case from Switzerland of severe fetal congenital toxoplasmosis that led to elective pregnancy termination due to severe CNS disease. Autopsy confirmed disseminated toxoplasmosis [10].

### 4.2. Reactivation of Chronic Latent Toxoplasma Infections vs. Acute Primary Infections

Toxoplasmosis cases can occur either due to reactivation of preexisting chronic latent *Toxoplasma* infections or due to acute primary *Toxoplasma* infections. Several of the toxoplasmosis cases associated with acute primary infections were severe, including cases with cerebral toxoplasmosis, disseminated disease, ocular toxoplasmosis with permanent vision loss or significant residual visual deficits [18,19,20,24,40,47,50], and a case of severe fetal congenital toxoplasmosis [10].

Among the cases associated with acute toxoplasma infections were three cases that occurred after consumption of undercooked wild game meat and undercooked other, not specified, type of meat while on targeted immunotherapies. These cases pertained to (a) a case with disseminated disease after eating wild boar sausages while on trametinib [5] [this patient eventually had clinical improvement]; (b) a case with bilateral ocular toxoplasmosis after consumption of undercooked venison meat while on erlotinib [26] (the patient eventually died 2 months later, not from toxoplasmosis, but likely from underlying metastatic lung adenocarcinoma) and (c) a case with severe chorioretinitis with ischemic necrotizing vasculitis after eating undercooked meat while on belatacept [unfortunately, this patient had no vision recovery despite anti-*Toxoplasma* treatment] [48]. The first two cases occurred in patients in the US.

### 4.3. Prevention of Toxoplasmosis

Toxoplasmosis in patients receiving targeted immunotherapies is a preventable condition. Screening for toxoplasmosis with *Toxoplasma* IgG and IgM (among other infectious agents) should be routinely carried out before initiating targeted immunotherapies. Moreover, routine education of all patients receiving targeted immunotherapies, to avoid exposure risks associated with toxoplasma infections through food or soil exposures, is critical [57]. Education for avoidance of consumption of undercooked meat and avoidance of wild game meat in particular can be lifesaving. Routine pre-screening ophthalmologic evaluation for evidence of old chorioretinal scars (that could reactivate during the targeted immunotherapy and lead to vision loss) is important. Primary anti-*Toxoplasma* prophylaxis should be considered for *T. gondii* IgG seropositive patients on targeted immunotherapies in order to prevent reactivation. These patients would also benefit from close clinical monitoring for signs and symptoms of reactivation and preemptive molecular laboratory monitoring (e.g., with blood *T. gondii* PCR) in the first 6–12 months after the initiation of targeted immunotherapies.

Acute *Toxoplasma* infections can be severe in these patients. Clinicians should be aware that *Toxoplasma* is a ubiquitous parasite and absence of classic risk factors for acute *Toxoplasma* infections [57] (e.g., eating undercooked meat, wild game meat, unwashed fruits and vegetables, drinking untreated water, exposure to soil through gardening or changing cat litter) should not exclude the diagnosis of toxoplasmosis if compatible clinical syndromes are present. Half of the acutely infected individuals do not report risk factors or behaviors associated with acute *Toxoplasma* infections [58].

### 4.4. High Index of Suspicion for Toxoplasmosis

Health care providers should consider toxoplasmosis in patients on biologics or small molecules presenting with encephalitis, altered mental status, cognitive impairment, seizures, motor deficits (hemiparesis [8], paraparesis [15], unsteady gait [6,8]), difficulties in fine motor coordination [6], cranial neuropathies (dysarthria, dysphagia), acute psychosis [16], myocarditis (with dyspnea, orthopnea, diffuse cardiac hypokinesia, high troponin) [6] pneumonia, myositis, hepatitis, lymphadenopathy or decreased visual acuity, as illustrated by this systematic review. Toxoplasmosis should also be considered in cases presenting with a tick-born-disease like illness (with leukopenia, lymphopenia, thrombocytopenia and transaminitis), or with pneumonia not responding to standard therapies [2,3].

Toxoplasmic chorioretinitis in patients on targeted immunotherapies can exhibit atypical features including bilateral involvement, extensive spread, multifocal presentation, large areas of retinal necrosis with or without adjacent retinal scar (in cases of reactivation or acute infections respectively), and diffuse necrotizing retinitis resembling viral retinitis [18]. Occasionally, residual permanent vision loss can occur in the affected eye [18,19,20,24,50].

Toxoplasmosis should be in the differential diagnosis in patients on biologics or small molecules, even if patients have been on anti-*Toxoplasma* prophylaxis. In this systematic review, breakthrough cases were reported, including after TMP/SMX [31,36] or azithromycin prophylaxis [35].

### 4.5. Diagnostics for Active Toxoplasmosis

Molecular diagnostics (PCR) are needed for the initial diagnosis of active toxoplasmosis and for early detection of relapses in cases associated with targeted immunotherapy with biologics or small molecules. Selection of the appropriate body fluid or tissue to test with *T. gondii* PCR is critical (e.g., blood, CSF, ocular or other body fluid/tissue). Parasitemia might not be present during all active toxoplasmosis cases (e.g., during ocular toxoplasmosis or during CNS disease). Testing of blood with agnostic metagenomic diagnostics, such as plasma cell-free DNA metagenomics next generation sequencing (e.g., Karius test [Karius Inc., Redwood City, CA, USA]) can help establish the diagnosis early in the course and can be life-saving, particularly when obtaining fluid or tissue biopsies (e.g., brain biopsy, CSF, or ocular fluid) is challenging or when the infectious diseases differential diagnosis is broad, as is the case in patients on targeted immunotherapies [43].

It is important to note that CSF analysis may be normal, and *T. gondii* PCR of CSF may be negative in some patients with cerebral toxoplasmosis, as has been shown even in cases where the diagnosis was confirmed by brain biopsy or autopsy [26,31,42].

Serology in the setting of reactivation may be difficult to interpret. It may only indicate positive *T. gondii* IgG, (with or without positive IgM) and the IgG avidity is usually high. The *T. gondii* IgG titers may be significantly elevated at the time of reactivation, and this important finding should not be erroneously dismissed as simply an indication of past infection (based on positive *T. gondii* IgG). Moreover, *T. gondii* serology may sometimes be completely negative in the setting of profound lymphopenia [34,53]. For acute primary *Toxoplasma* infections, attempts to retrieve serum samples prior to the onset of toxoplasmosis clinical presentation may be critical to help establish seroconversion (from *T. gondii* IgG negative status to IgG positive status) that would further support the diagnosis of acute infection. 

### 4.6. Modification of Biologics/Small Molecules Therapy During Active Toxoplasmosis

Modification of biologics or small molecules targeted immunotherapy in the setting of active toxoplasmosis may be required and should be made in consultation with a multidisciplinary team, including the primary treating specialty, clinical pharmacists, and infectious disease experts, particularly those specializing in immunocompromised hosts and toxoplasmosis. In the US, the Dr. Jack S. Remington Laboratory for Specialty Diagnostics, a non-profit laboratory and the CDC’s and FDA’s national reference center for toxoplasmosis in the US (https://www.sutterhealth.org/patient-resources/remington-laboratory, accessed on 18 September 2025), can assist in the management of those cases. Brief discontinuation of the biologic agent or small molecules, or at least a dosage decrease, may be necessary to reverse the immune defect that likely led to the reactivation of a latent infection or to a severe clinical presentation after an acute primary infection. If biologics or small molecules are decided to be continued (during or after the resolution of the active toxoplasmosis episode), close clinical monitoring and preemptive laboratory screening with molecular diagnostics, such as blood *T. gondii* PCR or plasma metagenomics tests, are needed to monitor treatment response and promptly recognize possible treatment failures or relapses.

In this systematic review, the continuation of the biologic or small molecule during active toxoplasmosis episode was successful in only two cases: a case of acute toxoplasmic lymphadenopathy [44] and a case of ocular toxoplasmosis [30]. In the case of toxoplasmic lymphadenopathy, the patient continued ixekizumab and had complete resolution after 14-days of pyrimethamine, leucovorin, and clindamycin with no recurrences during a five-year follow-up period [44]. In the case of ocular toxoplasmosis, the patient continued imatinib and the chorioretinal disease resolved within three months without recurrence [30]. In two other cases, there were complications. The first case pertained to a 17-year-old hematopoietic stem cell transplant recipient (HSCT) who developed cerebral toxoplasmosis while receiving ruxolitinib and in whom ruxolitinib treatment was continued at a reduced dose [38]. The patient demonstrated initial clinical improvement but subsequently developed Guillain-Barré Syndrome (GBS), possibly also attributed to toxoplasmosis. The second case pertained to a patient with severe psoriasis receiving ustekinumab, who presented with floaters in the right eye and was diagnosed with ocular toxoplasmosis [14]. The biologic was initially continued while the patient was treated for four weeks with oral pyrimethamine, azithromycin, folinic acid, and dexamethasone, resulting in complete symptom resolution. However, four months later, the patient experienced a clinical relapse complicated by irreversible vision loss in the affected eye due to foveal involvement. Following this relapse, ustekinumab was permanently discontinued.

### 4.7. Anti-Toxoplasma Treatment

Anti-*Toxoplasma* therapy is necessary for most cases of toxoplasmosis in patients receiving biologic agents or small molecules. Only one case of toxoplasmosis was successfully treated simply by discontinuing the biologic agent, without initiation of anti-*Toxoplasma* therapy. This case pertained to a 26-year-old male with psoriasis vulgaris on ustekinumab in whom the acute toxoplasmosis self-improved after discontinuation of the biologic agent [45]. This patient had a mononucleosis-like illness with generalized lymphadenopathy. After the three month follow up, the patient remained symptom-free, but a decision was made to permanently discontinue biologic therapy. 

### 4.8. Secondary Prophylaxis

After completion of anti-*Toxoplasma* therapy, and prior to reinitiating the biologic or small molecule, patients should either be placed on secondary anti-*Toxoplasma* prophylaxis or should remain under close clinical and preemptive laboratory surveillance with molecular diagnostics (e.g., with blood *T. gondii* PCR). 

### 4.9. Special Consideration

#### 4.9.1. For Pregnant Women on Biologics/Small Molecules Before Pregnancy

Special considerations are needed for women planning to become pregnant after having been on biologics or small molecules prior to conception. Immune dysregulation can persist for several months after discontinuation of these targeted immunotherapies, increasing the risk for vertical transmission in *T. gondii* seropositive women. 

This systematic review identified a case of severe fetal congenital toxoplasmosis [10], diagnosed at around 27 weeks gestational age, associated with a severely abnormal fetal cerebral ultrasound and brain MRI and positive amniotic fluid *T. gondii* PCR. An elective pregnancy termination was decided and autopsy confirmed disseminated congenital toxoplasmosis with necrotizing encephalitis, adrenal gland calcifications and hepatosplenomegaly. The mother of this fetus had discontinued adalimumab treatment for her ankylosing spondylitis five months prior to conception. Retrospectively, serology confirmed that the mother had an acute *Toxoplasma* infection 6–7 months prior to conception, while on adalimumab. Although the mean half-life of adalimumab is only two weeks (ranging from 10–20 days across studies [59]), the immunomodulatory effect likely persisted for more than six months despite discontinuation, leading to ineffective immune control of the toxoplasmic infection and presumed prolonged persistent parasitemia after the acute infection. Alternatively, local reactivation of *T. gondii* in the uterine wall after the prior acute infection may have contributed to the vertical transmission.

Women who plan to become pregnant, after having been on biologics or small molecules, should be screened for toxoplasmosis. If there is evidence of a prior *T. gondii* infection, these women should: (a) delay pregnancy for at least 6–12 months after stopping the biologics/small molecules, (b) undergo ophthalmologic evaluation for evidence of old toxoplasmic chorioretinal scars (that could reactivate during pregnancy and lead to vertical transmission) and (c) be screened with blood *T. gondii* PCR prior to becoming pregnant; and then (if blood PCR is negative), be placed on spiramycin prophylaxis throughout their future gestation to prevent vertical transmission. The immunomodulatory effects of biologic agents/small molecules likely persist for more than 6 months after their discontinuation. 

#### 4.9.2. For Patients on CAR-T Cell Therapies

Although neurotoxicity after CAR T-cells therapy is well documented, health care providers should be aware that cerebral toxoplasmosis can mimic the neurologic manifestations of post CAR T-cell neurotoxicity and prompt diagnostic evaluation for toxoplasmosis should be considered in cases with compatible clinical syndromes.

#### 4.9.3. Coinfections with Other Notable Pathogens

While biologics and small molecules may facilitate reactivation of multiple chronic/latent infections, this systematic review focused exclusively on toxoplasmosis. Nevertheless, we identified three cases that documented reactivation of additional pathogens concomitantly with the reactivation of toxoplasmosis. The first case had concurrently cerebral toxoplasmosis and progressive multifocal leukoencephalopathy (PML) due to JC virus reactivation in a chronic lymphocytic leukemia patient receiving rituximab [27]. The second case had severe cerebral toxoplasmosis along with low level EBV viremia in a patient with rheumatoid arthritis on anti-TNF alpha therapy (among other regimens) [15]. Despite low levels of EBV viremia, the neurologic findings in this case were clearly suggestive of cerebral toxoplasmosis, as confirmed by brain biopsy with detection of *Toxoplasma* tachyzoites and clinical response to anti-*Toxoplasma* therapy. The third case was a patient with rheumatoid arthritis on abatacept who developed bilateral granulomatous panuveitis with retinal necrosis and who, despite anti-*Toxoplasma* treatment, developed permanent vision loss in the right eye [20]. In this case, the vitreous fluid was PCR positive for both *T. gondii* and EBV.

### 4.10. Study Limitations

Some study limitations should be acknowledged. First, although this systematic review utilized broad search strategies targeting several classes of biologics and small molecules, including 106 individual biologics or small molecules (in addition to the general drug classes), some pertinent cases may have been missed. Second, this systematic review identified 26 cases of active toxoplasmosis that were co-attributed to the targeted immunotherapy among other immunosuppressants. In these cases, at the time of active toxoplasmosis diagnosis, the patients were receiving/or had recently received additional immunosuppressants. However, the fact that this systematic review also identified 20 cases of active toxoplasmosis after monotherapy with the targeted immunotherapies provides proof of concept for their causative role in the development of toxoplasmosis. Among the toxoplasmosis cases that occurred while on monotherapy with the targeted immunotherapy, 60% (n = 12) had acute toxoplasmosis, 20% (n = 4) had reactivation of disease; in the remaining 20% (n = 4), it was unclear. The clinical manifestation of toxoplasmosis in those cases was as follows: 40% (n = 8) had ocular toxoplasmosis, 30% (n = 6) cerebral toxoplasmosis, 15% (n = 3) lymphadenopathy, 5% (n = 1) disseminated toxoplasmosis, 5% (n = 1) pneumonic toxoplasmosis, and 5% (n = 1) lead to congenital toxoplasmosis. Notably, 55% (n = 11) of the cases on monotherapy (with targeted immunotherapy) resulted in severe manifestation of toxoplasmic disease, including six cases with cerebral disease, three with severe ocular disease resulting in permanent visual deficits, one with disseminated disease, and one with pneumonic disease. In these cases, the targeted immunotherapies were adalimumab, alemtuzumab, belatacept, erlotinib, etanercept, fingolimod, golimumab, imatinib, iscalimab, ixekizumab, natalizumab, rituximab, ruxolitinib, and ustekinumab. Third, this paper did not provide a detailed discussion of the pathophysiologic mechanisms explaining the contributing role of the targeted immunotherapies to the active toxoplasmosis; however, this was beyond the scope of this clinical systematic review.

## 5. Conclusions

In summary, healthcare providers should screen for *Toxoplasma* infection, among other infectious diseases, in patients planning to undergo treatment with biologics (including CAR T-Cell therapies) or small molecules. Primary anti-*Toxoplasma* prophylaxis should be considered in patients who are *T. gondii* IgG seropositive prior to starting the targeted immunotherapy. *Women of reproductive age who are planning to become pregnant after having been on biologics or small molecules, should delay conception for at least 6-12 months after discontinuation of the targeted immunotherapy and infectious diseases physicians, experts in toxoplasmosis in immunocompromised hosts, should be consulted for screening and management during pregnancy, including consideration of anti-Toxoplasma prophylaxis for IgG seropositive women.* Prompt education for avoidance of high-risk exposures (through food or soil) should be routinely implemented in these patients. Severe toxoplasmosis in this patient population can occur both from reactivation of chronic latent *Toxoplasma* infections and from acute primary *Toxoplasma* infections. A high index of suspicion for toxoplasmosis should be kept in patients on biologics or small molecules who develop clinical syndromes that are compatible with toxoplasmosis. Prompt diagnosis and treatment can be lifesaving. Modification or discontinuation of biologics or small molecules may be needed during active toxoplasmosis. Close clinical and molecular laboratory monitoring should be maintained after resolution of the active disease if targeted immunotherapy agents are to be re-instituted. Consultation with a multidisciplinary team, including the primary specialty team, infectious disease experts specializing in immunocompromised hosts and toxoplasmosis, and clinical pharmacists, are needed. Establishing registries of patients receiving biologics or small molecules for toxoplasmosis or other infectious diseases complications can deepen our understanding of the specific immune defects predisposing to these infections and inform the development of targeted prevention and treatment protocols.

## Figures and Tables

**Table 1:** Characteristics of toxoplasmosis cases after targeted immunotherapy with biologics or small molecules.

Total	Number of Cases (n = 46)	% (n = 46)	References
*Sex*			
Female	24	52%	
Male	21	46%	
Not Reported	1	2%	
*Indication for Targeted Immunotherapy-Condition*			
Rheumatologic	16	35%	[10,14,15,16,17,18,19,20,21,22,23,24,25,26]
Oncologic	12	26%	[27,28,29,30,31,32,33,34,35,36,37]
Transplant	5	11%	[38,39,40,41,42]
Dermatological autoimmune	5	11%	[43,44,45,46,47]
Gastrointestinal autoimmune	3	7%	[48,49,50]
Neurological autoimmune	3	7%	[51,52,53]
Pulmonary autoimmune	1	2%	[54]
Both Oncologic and Rheumatologic	1	2%	[6]
*Biologics*			
Anti TNF-α (e.g., adalimumab, etanercept, golimumab, infliximab)	18	39%	
Anti-CD20 (e.g., rituximab)	5	11%	
Anti-CD-52 (e.g., alemtuzumab)	4	9%	
T-cell co-stimulation inhibitor (e.g., abatacept, belatacept)	3	7%	
Anti-IL-12/IL-23 (e.g., ustekinumab)	2	4%	
Anti-IL-17 (e.g., ixekizumab)	1	3%	
Anti-cell adhesion molecule/integrin receptor inhibitor (e.g., natalizumab)	1	2%	
Anti-CD40 (e.g., iscalimab)	1	2%	
CAR T-cells	3	7%	
*Small Molecules*			
JAK inhibitors (e.g., ruxolitinib)	4	9%	
Tyrosine Kinase Inhibitor (e.g., erlotinib, imatinib)	2	4%	
MEK inhibitors (e.g., trametinib)	1	2%	
Sphingosine 1-phosphate receptor modulator (e.g., fingolimod)	1	2%	
*Duration of Targeted Immunotherapy Prior to the Onset of Toxoplasmosis (per Type of Toxoplasma Infections)*			
*In Acute Toxoplasma infections*			
3 weeks to >4 years	13	28%	
Not reported	5	11%	
*In Reactivations*			
1–2 months	6	13%	
>2 months	6	13%	
Not reported	7	15%	
Single dose only	1	2%	
*In Unclear Types of Infections*			
1 month to 4 years	6	13%	
Single dose only	*2*	*4%*	
*Attribution of Toxoplasmosis to Targeted Immunotherapy*			
*Clearly Attributed*	*25*	*54%*	
Targeted immunotherapy was given as monotherapy (n = 20)			[10,14,19,21,24,28,30,32,37,41,42,43,44,45,47,49,51,52,53,54]
Recent addition of a targeted immunotherapy to a preexisting regimen of immunosuppressants (n = 4)			[8,19,38,46]
Recent increase in the dose of targeted immunotherapy (n = 1)			[25]
*Likely at least co-attributed* Targeted immunotherapy was given along with other immunosuppressant(s) (n = 21)	*21*	*46%*	
*Type of Toxoplasma Infections*			
Reactivation of chronic latent infection	20	44%	[8,15,17,18,19,20,23,25,26,29,31,35,36,39,41,42,46,50,52]
Acute primary infection ^¥^	18	39%	[6,10,14,19,22,24,28,32,37,38,40,43,44,45,47,48,49,51]
Unclear/Not reported	8	17%	[16,17,21,27,30,34,53,54]
*Toxoplasmosis Clinical Syndromes* *			
Cerebral toxoplasmosis	23	50%	[8,15,16,17,21,23,25,26,27,29,31,34,35,36,38,39,41,43,46,51,53,54]
Ocular toxoplasmosis	15	33%	[18,19,20,22,24,28,30,37,40,47,48,49,50,52]
Lymphadenopathy	3	7%	[14,44,45]
Disseminated toxoplasmosis	2	4%	[6,33]
Cerebral and Ocular	1	2%	[42]
Pneumonic toxoplasmosis ^&^	1	2%	[32]
Congenital toxoplasmosis (abnormal fetal scan/elective termination of pregnancy)	1	2%	[10]

* Some patients had more than one clinical syndrome. ^¥^ Overall, in the setting of 18 acute toxoplasmosis cases, there were three cases of cerebral toxoplasmosis; nine cases of ocular toxoplasmosis three cases of lymphadenopathy; one case of disseminated disease, one case of pneumonic toxoplasmosis and one case of fetal congenital toxoplasmosis. ^&^ One additional case of pulmonic toxoplasmosis (with respiratory failure and ground glass opacities in CT chest) occurred in a patient with disseminated disease and multiorgan failure from toxoplasmosis [33].

**Table 2:** Toxoplasmosis manifestations according to targeted immunotherapy category.

*Biologics*		
Anti-CD20 inhibitors	Rituximab (n = 5)	Cerebral toxoplasmosis with space occupying lesions (n = 5) [16,27,29,43,46]
Anti-CD40 inhibitors	Iscalimab (n = 1)	Disseminated toxoplasmosis (n = 1) [42]
Anti-CD52 Inhibitors	Alemtuzumab (n = 4)	Cerebral toxoplasmosis (n = 3) [31,36,53]
Anti-IL12/IL23 inhibitors	Ustekinumab (n = 2)	Ocular toxoplasmosis-with vision loss (n = 1) [47] Lymphadenopathy (n = 1) [45]
Anti-IL-7A inhibitors	Ixekizumab (n = 1)	Lymphadenopathy [44]
Integrase inhibitors	Natalizumab (n = 1)	Ocular toxoplasmosis [52]
TNF-a inhibitors	Adalimumab (n = 11)	Lymphadenopathy/Malaise (n = 1) [14] Cerebral toxoplasmosis (n = 3) [8,21,26] Ocular toxoplasmosis (n = 6) [18,22,24,48,49,50] Congenital toxoplasmosis (n = 1) [10]
	Golimumab (n = 1)	Cerebral toxoplasmosis [54]
	Infliximab (n = 4)	Cerebral toxoplasmosis with space occupying lesions (n = 3) [17,23,25] Ocular toxoplasmosis with vision loss (n = 1) [19]
	TNF-a inhibitor (specific name not reported) (n = 1)	Cerebral toxoplasmosis with space occupying lesion (n = 1) [15]
TNF-a and b inhibitors	Etanercept (n = 1)	Ocular toxoplasmosis [19]
T-cell co-stimulation inhibitors	Belatacept (n = 2)	Cerebral toxoplasmosis (n = 1) [41] Ocular toxoplasmosis-with vision loss (n = 1) [40]
	Abatacept (n = 1)	Ocular toxoplasmosis [20]
CAR T-cells (n = 3)		Cerebral toxoplasmosis (n = 2) [34,35] Disseminated (n = 1) [33]
*Small Molecules*		
JAK inhibitors	Ruxolitinib (n = 3)	Cerebral toxoplasmosis-with space occupying lesions (n = 2) [38,39] Ocular toxoplasmosis-bilateral (n = 1) [28]
JAK 3 inhibitor	Specific name not reported (n = 1)	Pneumonic toxoplasmosis [32]
MEK inhibitors	Trametinib (n = 1)	Disseminated toxoplasmosis (cerebral, ocular, myocarditis, myositis) [6]
Tyrosine Kinase Inhibitor	Erlotinib (n = 1)	Ocular toxoplasmosis [37]
	Imatinib (n = 1)	Ocular toxoplasmosis [30]
Sphingosine 1-phosphate receptor modulator	Fingolimod (n = 1)	Cerebral toxoplasmosis [51]

**Table 3:** Management of Toxoplasmosis cases (Diagnosis, Therapy, Outcome, Secondary Prophylaxis).

		Number of Cases (n = 46)	% (n = 46)	References
***T. gondii* Diagnostics ^&^**				
*Serology*		*33*	*72%*	
Serology only		10		
Multimodal diagnostics with serology		23		
*PCR*		*21*	*46%*	
PCR only (CSF, blood, aqueous or vitreous fluid, amniotic fluid)		4		
Multimodal diagnostics with PCR		17		
*Brain Biopsy*		*15*	*33%*	
Brain biopsy histopathology only		2		
Multimodal diagnostics with brain biopsy		13		
*Radiological characteristics typical (negative serology)*		*2*	*4%*	
*Not reported*		*1*	*2%*	
*Multimodal T. gondii Diagnostics*		*27*	*59%*	
Serology and PCR of body fluid (CSF, blood, aqueous, or vitreous fluid)		12		
Serology and Next Generation Sequencing (plasma) [43]		1		
Serology and Brain biopsy histopathology/immunohistochemistry		9		
Brain biopsy histopathology/immunohistochemistry and PCR		4		
Serology, PCR (amniotic fluid), fetal autopsy		1		
***Modification of Targeted Immunotherapy at the time of Presentation of Toxoplasmosis***				
Discontinued (temporarily/deferred or permanently) *		25	54%	[8,14,15,17,18,19,20,21,22,24,26,28,29,39,41,42,43,45,47,48,49,50,51,52,53]
Continued		2	4%	[30,44]
Decreased		1	2%	[38]
Not applicable: Patient had completed targeted immunotherapy prior to infection; or had received CAR T-cells single infusion or patient had died or had discontinued targeted immunotherapy prior to conception		10	22%	[10,16,31,32,33,34,35,46,54]
Not reported		8	17%	
***Modification of Targeted Immunotherapy after completion of the anti-Toxoplasma treatment*** *(applicable only for the cases who had discontinued the targeted immunotherapy at the time of diagnosis) (n = 25)*				
Permanent discontinuation of biologic/small molecule		6		[8,15,24,29,43,48]
Permanent discontinuation of all immunosuppressants (including targeted immunotherapies)		3		[45,50,53]
Temporary discontinuation		2		[14,47]
Switch to another biologic/small molecule		2		[22,51]
Not reported		12		[17,18,19,20,21,26,28,39,41,42,49,52]
***Initial Anti-Toxoplasma Therapy***				
Pyrimethamine + Sulfadiazine + Folinic acid		18	39%	[8,15,16,19,21,29,31,32,34,35,40,43,46,49,52,53]
TMP-SMX		17	37%	[6,14,17,18,20,22,24,26,28,30,33,39,41,42,48,50,54]
Pyrimethamine + Other agents (e.g., clindamycin, azithromycin, dapsone)		6	13%	[23,25,38,44,47,51]
Atovaquone		1	2%	[37]
No treatment (only discontinuation of the Biologic agent)		1	2%	[45]
No treatment (Case of elective pregnancy termination due to severe Congenital Toxoplasmosis)		1	2%	[10]
Not reported		2	4%	[27,36]
***Duration of Anti-Toxoplasma Therapy***				
2–3 weeks		1	2%	[44]
4–5 weeks		5	11%	[24,47,49,52,53]
6–8 weeks		13	28%	[8,17,19,20,21,22,29,30,32,35,40,41,42]
10–16 weeks		4	9%	[6,15,39,43]
No treatment—only discontinuation of biologic agent		1	2%	[45]
No treatment—elective pregnancy termination due to severe congenital toxoplasmosis, from a retrospectively identified acute maternal *Toxoplasma* infection 6–7 months before conception)		1	2%	[10]
Not applicable—patient expired		3	7%	[31,33,54]
Not reported		18	39%	
***Clinical Outcomes***				
*Favorable Outcomes*		*27*	*59%*	
Complete Recovery	Complete clinical recovery (n = 7)	9	20%	[8,14,30,39,44,45,53]
	Complete radiographic recovery (n = 2)			[36,43]
Clinical Improvement (by the time of last follow up)		17	37%	[6,15,19,21,22,23,25,28,29,31,34,35,41,42,48,49,52]
Radiographic improvement		1	2%	[51]
*Unfavorable Outcomes*		*17*	*37%*	
Residual deficits or no recovery	Cerebral (n = 1)	8	17%	[46]
	Ocular (n = 7)			[18,19,20,24,40,47,50]
Complications	Guillain Barre Syndrome (GBS) ^#^ [38]; Brain hemorrhage after brain biopsy ^Δ^ [17]; Psychiatric complications [16]	3	7%	[16,17,38]
Deceased due to toxoplasmosis		4	9%	[27,31,33,54]
Deceased not due to toxoplasmosis (likely from metastatic lung adenocarcinoma after clinical improvement of ocular toxoplasmosis)		1	2%	[37]
Terminated pregnancy (due to severe congenital toxoplasmosis in the fetus)		1	2%	[10]
*Not reported*		*2*	*4%*	[26,36]
***Secondary Prophylaxis***				
TMP/SMX:	Cerebral toxoplasmosis	7	15%	[8,39,43]
	Ocular toxoplasmosis			[18,20]
	Pneumonic toxoplasmosis			[32]
	Cerebral and ocular toxoplasmosis			[42]
Pyrimethamine/Sulfadiazine/Folinic acid:	Cerebral toxoplasmosis	3	7%	[29,35,53]
Atovaquone:	Cerebral toxoplasmosis	1	2%	[15]
No prophylaxis:	Ocular	1	3%	[22]
Not applicable:	Congenital toxoplasmosis	5	11%	[10]
	Expired from cerebral toxoplasmosis			[27,31,54]
	Expired from disseminated toxoplasmosis			[33]
Not reported		29	63%	

^&^ In some cases, more than one diagnostic was used; thus, the total may exceed 100%. * Includes a patient who initially continued biologic therapy but had a toxoplasmosis recurrence 4 months after the initial episode, and thus the biologic agent (ustekinumab) was permanently discontinued [15]. ^#^ Represents a case of Guillain–Barré syndrome, likely also associated with toxoplasmosis. ^Δ^ Brain hemorrhage after the brain biopsy for the diagnosis of cerebral toxoplasmosis.

## Data Availability

All data for this project are available in the main manuscript, figures, tables, and Supplementary Materials.

## Supplementary Materials

The following supporting information can be downloaded at: https://www.mdpi.com/article/10.3390/pathogens14101001/s1. Table S1: Search strategies; Table S2: List of the 106 individual biologic agents or small molecules searched (in addition to drug classes) in the listed PubMed search strategies; Table S3: PRISMA checklist and PRISMA 2020 for Abstracts Checklist; Table S4: List of 44 articles (for the 46 cases) included in the systematic review; Table S5: Compilation of clinical vignettes of the 46 toxoplasmosis cases in patients on biologics or small molecules for autoimmune, oncologic or transplant conditions. Table S6: Raw data extracted from the 46 cases.
