# Supplementary material for: Toxoplasmosis in the Era of Targeted Immunotherapy: A Systematic Review of Emerging Cases Linked to Biologics and Small Molecules in Autoimmune Diseases, Oncology and Transplantation
Source: Pathogens. 2025 Oct 3;14(10):1001. doi: 10.3390/pathogens14101001 (PMC12567518; doi:10.3390/pathogens14101001)
Supplement: Supplementary file 1 [file pathogens-14-01001-s001.zip › pathogens-3846958 supplementary material_TablesS1_S5_09252025-0926_SC.pdf]

SUPPLEMENTARY MATERIAL
Tables S1-S5

Table S1: Search strategies

We performed two PubMed Searches.
In the initial search, we searched PubMed up to 1/1/2025 using the following search strategy: **Search #1:** (Toxoplasm* OR T. gondii) AND (TNF inhibitors OR Etanercept OR Infliximab OR Adalimumab OR Certolizumab OR Golimumab OR IL-1 inhibitors OR Anakinra OR Canakinumab OR Rilonacept OR IL-6 inhibitors OR Tocilizumab OR Sarilumab OR IL-17 inhibitors OR Secukinumab OR Ixekizumab OR Bimekizumab OR Brodalumab OR IL-12/23 Blockade OR Ustekinumab OR Guselkumab OR Risankizumab OR Costimulation blockade OR Abatacept OR (B cell depletion OR B cell Inhibition) OR Rituximab OR Belimumab OR Anifrolumab OR Janus Kinase Inhibitors OR Baricitinib OR Tofacitinib OR Upadacitinib OR Filgotinib OR Peficitinib OR Ruxolitinib OR Abrocitinib OR Ritlecitinib OR Deucravacitinib OR Immune checkpoint inhibitors OR Nivolumab OR Pembrolizumab OR Cemiplimab OR Dostarlimab OR Retifanlimab OR Toripalimab OR Tislelizumab OR Atezolizumab OR Avelumab OR Durvalumab OR Ipilimumab OR Tremelimumab).
SEARCH RESULTS from search #1:
We retrieved 153 articles from this search strategy, and 30 articles (31 cases) were considered eligible for inclusion.

We updated our search up to 7/19/2025, using a more extended search strategy, using also as filter "case reports or observational studies". The updated search strategy was the following:
Search #2: (Toxoplasm* OR T.gondii) AND (Monoclonal antibod* OR TNF inhibitor* OR TNF-inhibitor* OR Adalimumab OR Certolizumab OR Etanercept OR Golimumab OR Infliximab OR Denosumab OR Omalizumab OR Ranibizumab OR Trastuzumab OR Eculizumab CD20 inhibitor* OR Anifrolumab OR Belimumab OR Obinutuzumab OR Ocrelizumab OR Ofatumumab OR Rituximab OR Ublituximab OR CD52 inhibitor* OR Alemtuzumab OR Checkpoint Inhibitor* OR Atezolizumab OR Avelumab OR Cemiplimab OR Dostarlimab OR Durvalumab OR Ipilimumab OR Nivolumab OR Pembrolizumab OR Relatimab OR Retifanlimab OR Tislelizumab OR Toripalimab OR Tremelimumab OR Co-stimulant inhibitor* OR Abatacept OR Belatacept OR FGFR inhibitor* OR Futibatinib OR IL1 inhibitor* OR IL-1 inhibitor* OR Anakinra OR Canakinumab OR Rilonacept OR IL12/IL23 inhibitor* OR IL-12/IL-23 inhibitor*OR Guselkumab OR Risankizumab OR Ustekinumab OR IL 17 inhibitor* OR IL-17 inhibitor* OR Bimekizumab OR Brodalumab OR Ixekizumab OR Secukinumab OR IL2 inhibitor* OR IL-2 inhibitor* OR Basiliximab OR IL4 inhibitor* OR IL-4 inhibitor* OR Dupilumab OR IL6 inhibitor* OR IL-6 inhibitor* OR Sarilumab OR Tocilizumab OR Integrin inhibitor* OR Eptifibatide OR Etrolizumab OR Natalizumab OR Tirofiban OR Vedolizumab OR chimeric antigen receptor T-cell OR chimeric antigen receptor T-cell OR chimeric antigen receptor* OR (CAR-T) OR (CAR T) OR (CAR T cell therapy) OR (CAR T-cell) OR ((CAR) T-cell) OR Kinase inhibitor* OR Ensartinib OR Acalabrutinib OR Ibrutinib OR Pirtobrutinib OR Zanubrutinib OR Momelotinib OR Pacritinib OR Abrocitinib OR Baricitinib OR Deucravacitinib OR Decernotinib OR Dilmapimod OR Evobrutinib OR Fostamatinib OR Filgotinib OR Pamapimod OR Iguratimod OR Peficitinib OR Ritlecitinib OR Ruxolitinib OR Tofacitinib OR Upadacitinib OR Capivasertib OR Lazertinib OR Abemaciclib OR Afatinib OR Axitinib OR Bosutinib OR Cabozantinib OR Dabrafenib OR Erlotinib OR Gefitinib OR Imatinib OR Lapatinib OR Levatinib OR Nilotinib OR Nintedanib OR Osimertinib OR Pegaptanib OR Ponatinib OR Ruxolitinib OR Sorafenib OR Spebrutinib OR Sunitinib OR Tirabrutinib OR Tovorafenib OR Vandetanib OR Vemurafenib OR Vorasidenib OR Duvelisib OR Idelalisib OR Inavolisib OR Repotrectinib OR Revumenib OR VEGF inhibitor* OR VEGF-inhibitor* OR Aflibercept OR Bevacizumab OR VEGFR inhibitor* OR VEGFR-inhibitor* OR Fruquintinib OR CDK 4/6 inhibitor* OR CDK4/6 inhibitor* OR Ribociclib OR Fingolimod).
SEARCH RESULTS from Search #2

We retrieved 93 articles, and 14 new articles were considered eligible for inclusion.

ADDITIONAL SEARCHES

We also screened the reference list of the included articles and 3 additional cases from 2 articles were identified

SUMMARY RESULTS:

In total 44 eligible papers (46 eligible cases) were analyzed.

Table S2: List of the 106 individual biologic agents or small molecules searched (in addition to drug classes) in the listed PubMed search strategies

	Targeted Immunotherapy	Class	Subclass
1	Abatacept	Co-stimulation inhibitors	Co-stimulation inhibitors
2	Abemaciclib	Kinase inhibitors	Kinase inhibitors
3	Abrocitinib	Kinase inhibitors	Janus Kinase Inhibitor
4	Acalabrutinib	Kinase inhibitors	BTK inhibitors
5	Adalimumab	TNF inhibitor	TNF inhibitor
6	Afatinib	Kinase inhibitors	Kinase inhibitors
7	Aflibercept	VEGF inhibitor	VEGF inhibitor
8	Alemtuzumab	CD52 inhibitor	CD52 inhibitor
9	Atezolizumab	Checkpoint inhibitors	Checkpoint inhibitors
10	Anakinra	IL1 inhibitor	IL1 inhibitor
11	Anifrolumab	CD20inhibitor	CD20inhibitor
12	Avelumab	Checkpoint inhibitors	Checkpoint inhibitors
13	Axitinib	Kinase inhibitors	Kinase inhibitors
14	Baricitinib	Kinase inhibitors	Janus Kinase Inhibitor
15	Basiliximab	IL-2 inhibitor	IL-2 inhibitor
16	Belatacept	Co-stimulation inhibitors	Co-stimulation inhibitors
17	Belimumab	CD20inhibitor	CD20inhibitor
18	Bevacizumab	VEGF inhibitor	VEGF inhibitor
19	Bimekizumab	IL17 inhibitor	IL17 inhibitor
20	Bosutinib	Kinase inhibitors	Kinase inhibitors
21	Brodalumab	IL17 inhibitor	IL17 inhibitor
22	Cabozantinib	Kinase inhibitors	Kinase inhibitors
23	Canakinumab	IL1 inhibitor	IL1 inhibitor
24	Capivasertib	Kinase inhibitors	Kinase inhibitor
25	CAR T-cells	CAR T-cell therapy	CAR T-cell therapy
26	Cemiplimab	Checkpoint inhibitors	Checkpoint inhibitors
27	Certolizumab	TNF inhibitor	TNF inhibitor
28	Dabrafenib	Kinase inhibitors	Kinase inhibitors
29	Denosumab	monoclonal antibody	monoclonal antibody
30	Deucravacitinib	Kinase inhibitors	Janus Kinase Inhibitor
31	Dostarlimab	Checkpoint inhibitors	Checkpoint inhibitors
32	Durvalumab	Checkpoint inhibitors	Checkpoint inhibitors
33	Duvelisib	Kinase inhibitors	PI3K inhibitor
34	Dupilumab	IL4 inhibitors	IL4 inhibitors
35	Eculizumab	Anti-complement 5	Anti-complement 5
36	Ensartinib	Kinase inhibitors	ALK inhibitor
37	Eptifibatide	Integrin inhibitor	Integrin inhibitor
38	Erlotinib	Kinase inhibitors	Kinase inhibitors
39	Etanercept	TNF inhibitor	TNF inhibitor
40	Etrolizumab	Integrin inhibitor	Integrin inhibitor
41	Filgotinib	Kinase inhibitors	Janus Kinase Inhibitor
42	Fingolimob	small molecule for MS Tx	small molecule for MS Tx
43	Fruquintinib	VEGFR inhibitor	VEGFR inhibitor
44	Futibatinib	FGFR inhibitor	FGFR inhibitor

45	Gefitinib	Kinase inhibitors	Kinase inhibitors
46	Golimumab	TNF inhibitor	TNF inhibitor
47	Guselkumab	IL12/23inhibitor	IL12/23inhibitor
48	Ibrutinib	Kinase inhibitors	BTK inhibitors
49	Idelalisib	Kinase inhibitors	PI3K inhibitor
50	Imatinib	Kinase inhibitors	Kinase inhibitors
51	Inavolisib	Kinase inhibitors	PI3K inhibitor
52	Infliximab	TNF inhibitor	TNF inhibitor
53	Ipilimumab	Checkpoint inhibitors	Checkpoint inhibitors
54	Ixekizumab	IL17 inhibitor	IL17 inhibitor
55	Lapatinib	Kinase inhibitors	Kinase inhibitors
56	Lazertinib	Kinase inhibitors	Kinase inhibitor
57	Levatinib	Kinase inhibitors	Kinase inhibitors
58	Momelotinib	Kinase inhibitors	JAK inhibitor
59	Natalizumab	Integrin inhibitor	Integrin inhibitor
60	Nilotinib	Kinase inhibitors	Kinase inhibitors
61	Nintedanib	Kinase inhibitors	Kinase inhibitors
62	Nivolumab	Checkpoint inhibitors	Checkpoint inhibitors
63	Obinutuzumab	CD20inhibitor	CD20inhibitor
64	Ocrelizumab	CD20inhibitor	CD20inhibitor
65	Ofatumumab	CD20inhibitor	CD20inhibitor
66	Omalizumab	monoclonal antibody	monoclonal antibody
67	Osimertinib	Kinase inhibitors	Kinase inhibitors
68	Pacritinib	Kinase inhibitors	JAK2 inhibitor
69	Peficitinib	Kinase inhibitors	Janus Kinase Inhibitor
70	Pegaptanib	Kinase inhibitors	Kinase inhibitors
71	Pembrolizumab	Checkpoint inhibitors	Checkpoint inhibitors
72	Pirtobrutinib	Kinase inhibitors	BTK inhibitors
73	Ponatinib	Kinase inhibitors	Kinase inhibitors
74	Quizartinib	Kinase inhibitors	FLT3 (Feline McDonoughSarcoma-like tyrosine kinase)
75	Ranibizumab	monoclonal antibody	monoclonal antibody
76	Relatimab	Checkpoint inhibitors	Checkpoint inhibitors
77	Repotrectinib	Kinase inhibitors	TKI
78	Retifanlimab	Checkpoint inhibitors	Checkpoint inhibitors
79	Revumenib	Menin inhibitor	Menin inhibitor
80	Ribociclib	Kinase inhibitor	CDK4/6 inhibitor
81	Rilonacept	IL1 inhibitor	IL1 inhibitor
82	Risankizumab	IL12/23inhibitor	IL12/23inhibitor
83	Ritlecitinib	Kinase inhibitors	Janus Kinase Inhibitor
84	Rituximab	CD20inhibitor	CD20inhibitor
85	Ruxolitinib	Kinase inhibitors	Janus Kinase Inhibitor
86	Ruxolitinib	Kinase inhibitors	Kinase inhibitors
87	Sarilumab	IL6 inhibitors	IL6 inhibitors
88	Secukinumab	IL17 inhibitor	IL17 inhibitor
89	Sorafenib	Kinase inhibitors	Kinase inhibitors
90	Sunitinib	Kinase inhibitors	Kinase inhibitors
91	Tirofiban	Integrin inhibitor	Integrin inhibitor

92	Tislelizumab	Checkpoint inhibitors	Checkpoint inhibitors
93	Tocilizumab	IL6 inhibitors	IL6 inhibitors
94	Tofacitinib	Kinase inhibitors	Janus Kinase Inhibitor
95	Toripalimab	Checkpoint inhibitors	Checkpoint inhibitors
96	Tovorafenib	Kinase inhibitors	Kinase inhibitors
97	Trastuzumab	monoclonal antibody	monoclonal antibody
98	Tremelimumab	Checkpoint inhibitors	Checkpoint inhibitors
99	Ublituximab	CD20inhibitor	CD20inhibitor
100	Upadacitinib	Kinase inhibitors	Janus Kinase Inhibitor
101	Ustekinumab	IL12/23inhibitor	IL12/23inhibitor
102	Vandetanib	Kinase inhibitors	Kinase inhibitors
103	Vedolizumab	Integrin inhibitor	Integrin inhibitor
104	Vemurafenib	Kinase inhibitors	Kinase inhibitors
105	Vorasidenib	Kinase inhibitors	Kinase inhibitors
106	Zanubrutinib	Kinase inhibitors	BTK inhibitors

Table S3: PRISMA checklist and PRISMA 2020 for Abstracts Checklist

Table S3a: PRISMA checklist

Section and Topic	Item #	Checklist item	Location where item is reported
**TITLE**			
Title	1	Identify the report as a systematic review.	Page 1
**ABSTRACT**			
Abstract	2	See the PRISMA 2020 for Abstracts checklist.	Page 1
**INTRODUCTION**			
Rationale	3	Describe the rationale for the review in the context of existing knowledge.	Page 2
Objectives	4	Provide an explicit statement of the objective(s) or question(s) the review addresses.	Page 3
**METHODS**			
Eligibility criteria	5	Specify the inclusion and exclusion criteria for the review and how studies were grouped for the syntheses.	Page 2
Information sources	6	Specify all databases, registers, websites, organisations, reference lists and other sources searched or consulted to identify studies. Specify the date when each source was last searched or consulted.	Page 2
Search strategy	7	Present the full search strategies for all databases, registers and websites, including any filters and limits used.	Supplementary Table 1
Selection process	8	Specify the methods used to decide whether a study met the inclusion criteria of the review, including how many reviewers screened each record and each report retrieved, whether they worked independently, and if applicable, details of automation tools used in the process.	Page 2<br>Page 3, Figure 1
Data collection process	9	Specify the methods used to collect data from reports, including how many reviewers collected data from each report, whether they worked independently, any processes for obtaining or confirming data from study investigators, and if applicable, details of automation tools used in the process.	Page 3
Data items	10a	List and define all outcomes for which data were sought. Specify whether all results that were compatible with each outcome domain in each study were sought (e.g. for all measures, time points, analyses), and if not, the methods used to decide which results to collect.	Page 2
	10b	List and define all other variables for which data were sought (e.g. participant and intervention characteristics, funding sources). Describe any assumptions made about any missing or unclear information.	n/a (case reports)
Study risk of bias assessment	11	Specify the methods used to assess risk of bias in the included studies, including details of the tool(s) used, how many reviewers assessed each study and whether they worked independently, and if applicable, details of automation tools used in the process.	n/a (case report)
Effect measures	12	Specify for each outcome the effect measure(s) (e.g. risk ratio, mean difference) used in the synthesis or presentation of results.	n/a (only descriptives)
Synthesis methods	13a	Describe the processes used to decide which studies were eligible for each synthesis (e.g. tabulating the study intervention characteristics and comparing against the planned groups for each synthesis (item #5)).	Page 3
	13b	Describe any methods required to prepare the data for	n/a (only descriptives)

Section and Topic	Item #	Checklist item	Location where item is reported
		presentation or synthesis, such as handling of missing summary statistics, or data conversions.	
	13c	Describe any methods used to tabulate or visually display results of individual studies and syntheses.	Page 3
	13d	Describe any methods used to synthesize results and provide a rationale for the choice(s). If meta-analysis was performed, describe the model(s), method(s) to identify the presence and extent of statistical heterogeneity, and software package(s) used.	n/a
	13e	Describe any methods used to explore possible causes of heterogeneity among study results (e.g. subgroup analysis, meta-regression).	n/a
	13f	Describe any sensitivity analyses conducted to assess robustness of the synthesized results.	n/a
Reporting bias assessment	14	Describe any methods used to assess risk of bias due to missing results in a synthesis (arising from reporting biases).	n/a
Certainty assessment	15	Describe any methods used to assess certainty (or confidence) in the body of evidence for an outcome.	n/a
**RESULTS**			
Study selection	16a	Describe the results of the search and selection process, from the number of records identified in the search to the number of studies included in the review, ideally using a flow diagram.	Page 3; Figure 1
	16b	Cite studies that might appear to meet the inclusion criteria, but which were excluded, and explain why they were excluded.	Figure 1
Study characteristics	17	Cite each included study and present its characteristics.	Supplementary Table 4,5,6
Risk of bias in studies	18	Present assessments of risk of bias for each included study.	n/a
Results of individual studies	19	For all outcomes, present, for each study: (a) summary statistics for each group (where appropriate) and (b) an effect estimate and its precision (e.g. confidence/credible interval), ideally using structured tables or plots.	(a) Table 1-3; Page 3-15<br>(b) n/a
Results of syntheses	20a	For each synthesis, briefly summarise the characteristics and risk of bias among contributing studies.	n/a
	20b	Present results of all statistical syntheses conducted. If meta-analysis was done, present for each the summary estimate and its precision (e.g. confidence/credible interval) and measures of statistical heterogeneity. If comparing groups, describe the direction of the effect.	n/a
	20c	Present results of all investigations of possible causes of heterogeneity among study results.	n/a
	20d	Present results of all sensitivity analyses conducted to assess the robustness of the synthesized results.	n/a
Reporting biases	21	Present assessments of risk of bias due to missing results (arising from reporting biases) for each synthesis assessed.	n/a
Certainty of evidence	22	Present assessments of certainty (or confidence) in the body of evidence for each outcome assessed.	n/a
**DISCUSSION**			
Discussion	23a	Provide a general interpretation of the results in the context of other evidence.	Page 15-21

Section and Topic	Item #	Checklist item	Location where item is reported
	23b	Discuss any limitations of the evidence included in the review.	Page 20
	23c	Discuss any limitations of the review processes used.	Page 20
	23d	Discuss implications of the results for practice, policy, and future research.	Page 20-21
**OTHER INFORMATION**			
Registration and protocol	24a	Provide registration information for the review, including register name and registration number, or state that the review was not registered.	Stephanie Cho, Jose G Montoya, Despina Contopoulos-Ioannidis, Registration ID: https://osf.io/jz2fy/
	24b	Indicate where the review protocol can be accessed, or state that a protocol was not prepared.	Supplementary material
	24c	Describe and explain any amendments to information provided at registration or in the protocol.	n/a
Support	25	Describe sources of financial or non-financial support for the review, and the role of the funders or sponsors in the review.	Page 21
Competing interests	26	Declare any competing interests of review authors.	Page 21
Availability of data, code and other materials	27	Report which of the following are publicly available and where they can be found: template data collection forms; data extracted from included studies; data used for all analyses; analytic code; any other materials used in the review.	Text, Tables, and Supplementary material (include all summary and raw data)

From: Page MJ, McKenzie JE, Bossuyt PM, Boutron I, Hoffmann TC, Mulrow CD, et al. The PRISMA 2020 statement: an updated guideline for reporting systematic reviews. BMJ 2021;372:n71. doi: 10.1136/bmj.n71. This work is licensed under CC BY 4.0. To view a copy of this license, visit https://creativecommons.org/licenses/by/4.0/

Abbreviations: n/a (not applicable; no quantitative meta-analysis was performed, only summary statistics and descriptives were provided)

Table S3b: PRISMA 2020 for Abstracts Checklist

Section and Topic	Item #	Checklist item	Reported (Yes/No)
**TITLE**			
Title	1	Identify the report as a systematic review.	Yes
**BACKGROUND**			
Objectives	2	Provide an explicit statement of the main objective(s) or question(s) the review addresses.	Yes
**METHODS**			
Eligibility criteria	3	Specify the inclusion and exclusion criteria for the review.	Yes
Information sources	4	Specify the information sources (e.g. databases, registers) used to identify studies and the date when each was last searched.	
Risk of bias	5	Specify the methods used to assess risk of bias in the included studies.	n/a
Synthesis of results	6	Specify the methods used to present and synthesise results.	Yes
**RESULTS**			
Included studies	7	Give the total number of included studies and participants and summarise relevant characteristics of studies.	Yes

Section and Topic	Item #	Checklist item	Reported (Yes/No)
Synthesis of results	8	Present results for main outcomes, preferably indicating the number of included studies and participants for each. If meta-analysis was done, report the summary estimate and confidence/credible interval. If comparing groups, indicate the direction of the effect (i.e. which group is favoured).	Yes
**DISCUSSION**			
Limitations of evidence	9	Provide a brief summary of the limitations of the evidence included in the review (e.g. study risk of bias, inconsistency and imprecision).	n/a
Interpretation	10	Provide a general interpretation of the results and important implications.	Yes
**OTHER**			
Funding	11	Specify the primary source of funding for the review.	No (included in page 1)
Registration	12	Provide the register name and registration number.	Yes (page 2)

Table S4: List of 44 articles (for the 46 cases) included in the systematic review.

1. Azevedo, V. F., et al. (2010). "[Acute toxoplasmosis infection in a patient with ankylosing spondylitis treated with adalimumab: a case report]." Reumatismo 62(4): 283-285.
2. Bach, M., et al. (2020). "[Neurological symptoms in a patient on anti-TNF therapy, methotrexate and prednisolone for rheumatoid arthritis]." Internist (Berl) 61(3): 313-320.
3. Basu S, Das T, Biswas G. (2010). Bilateral toxoplasma retinochoroiditis in a patient with chronic myeloid leukemia treated with imatinib mesylate. Ocul Immunol Inflamm. 18(1):64-5.
4. Biancardi, A. L., et al. (2020). "Severe Necrotising Toxoplasmic Retinochoroiditis in a Patient With Crohn's Disease in Use of Adalimumab and Azathioprine." Inflamm Bowel Dis 26(7): e69-e70.
5. Castaño-Amores, C. and P. Nieto-Gomez (2021). "Cerebral toxoplasmosis associated with treatment with rituximab, azathioprine and prednisone for dermatomyositis." Br J Clin Pharmacol 87(3): 1525-1528.
6. Cren JB, Bouvard B, Crochette N. (2016). Cerebral toxoplasmosis and anti-TNFα: a case report. IDCases. 7;5:40-2.
7. de Almeida, G. B., et al. (2022). "Cerebral Toxoplasmosis as an Uncommon Complication of Biologic Therapy for Rheumatoid Arthritis: Case Report and Review of the Literature." Brain Sci 12(8).
8. Desmond, R., et al. (2010). "Progressive multifocal leukoencephalopathy and cerebral toxoplasmosis in a patient with CLL." Am J Hematol 85(8): 607.
9. Enriquez-Marulanda A, et al. (2017). Cerebral toxoplasmosis in an MS patient receiving Fingolimod. Mult Scler Relat Disord.18:106-108.
10. Gharamti, A. A., et al. (2018). "Acute Toxoplasma Dissemination With Encephalitis in the Era of Biological Therapies." Open Forum Infect Dis 5(11): ofy259.
11. Goldberg, R. A., et al. (2013). "Bilateral toxoplasmosis retinitis associated with ruxolitinib." N Engl J Med 369(7): 681-683.
12. Gonzalez Vicent, M., et al. (2019). "Toxoplasmosis and secondary Guillain-Barre associated with ruxolitinib as graft-versus-host disease treatment." Pediatr Blood Cancer 66(1): e27446.
13. Hill, B., et al. (2020). "Cerebral Toxoplasmosis in a Rheumatoid Arthritis Patient on Immunosuppressive Therapy." Cureus 12(6): e8547.
14. Hoellinger, B., et al. (2021). "Unusual presentation of toxoplasmosis with gastro-intestinal involvement in HLA non-identical stem cell transplantation." Transpl Infect Dis 23(4): e13616.
15. Javadzadeh, S., et al. (2020). "Ocular toxoplasmosis in a patient treated with ustekinumab for psoriasis." Clin Exp Dermatol 45(6): 802-804.
16. Kator S, et al. (2020). Disseminated toxoplasmosis and haemophagocytic lymphohistiocytosis following chimeric antigen receptor T-cell therapy. Br J Haematol. 189(1):e4-e6.
17. Kayabaşı, M., et al. (2024). "Active toxoplasma chorioretinitis in immunocompromised patients: a case series." Arch Clin Cases 11(1): 5-12.
18. Kersten MJ, van Ettekoven CN, Heijink DM. (2019). Unexpected neurologic complications following a novel lymphoma treatment 'expected' to give rise to neurologic toxicity. BMJ Case Rep. 12;12(11):e229946.
19. Krull, E., et al. (2021). "Congenital toxoplasmosis after adalimumab treatment before pregnancy." J Obstet Gynaecol Res 47(11): 4055-4059.
20. Lanfranco, L., et al. (2016). "Late isolated ocular toxoplasmosis in a belatacept-treated kidney transplant patient." Transpl Int 29(12): 1352-1353.
21. Lassoued, S., et al. (2007). "Toxoplasmic chorioretinitis and antitumor necrosis factor treatment in rheumatoid arthritis." Semin Arthritis Rheum 36(4): 262-263.
22. Lee, E. B., et al. (2020). "Cerebral toxoplasmosis after rituximab for pemphigus vulgaris." JAAD Case Rep 6(1): 37-41.
23. Lim Z, et al. (2007). Toxoplasmosis following alemtuzumab based allogeneic haematopoietic stem cell transplantation. J Infect. 54(2):e83-6.
24. Lobo, Y., et al. (2020). "Toxoplasmosis in a patient receiving ixekizumab for psoriasis." JAAD Case Rep 6(3): 204-206.

25. Martin SI, Marty FM, Fiumara K, Treon SP, Gribben JG, Baden LR. (2006) Infectious complications associated with alemtuzumab use for lymphoproliferative disorders. Clin Infect Dis. 43(1):16-24.
26. Martina MN, et al. (2011). Toxoplasma gondii primary infection in renal transplant recipients. Two case reports and literature review. Transpl Int. 24(1):e6-12.
27. Marzolini MAV, et al. (2019). Toxoplasmosis initially presenting as neurological sequelae of chimeric antigen receptor T-cell therapy. Lancet Infect Dis. 19(7):788.
28. Mejia-Salgado, G., et al. (2024). "Coinfection Suspicion is Imperative in Immunosuppressed Patients with Suspected Infectious Uveitis and Inadequate Treatment Response: A Case Report." Ocul Immunol Inflamm 32(10): 2548-2552.
29. Misra, D. P., et al. (2016). "Sarcoidosis, neurotoxoplasmosis and golimumab therapy." QJM 109(12): 817-818.
30. Muslimani, M. A. and J. Di Palma-Grisi (2019). "Severe acute toxoplasmosis infection following ustekinumab treatment in a patient with psoriasis vulgaris." BMJ Case Rep 12(8).
31. Nardone, R., et al. (2014). "Cerebral toxoplasmosis following adalimumab treatment in rheumatoid arthritis." Rheumatology (Oxford) 53(2): 284.
32. Patnaik, G., et al. (2024). "Ocular toxoplasmosis following anti-tumour necrosis factor-alpha therapy combined with oral methotrexate therapy: A case report and review of literature." Eur J Ophthalmol 34(2): NP113-NP117.
33. Pulivarthi, S., et al. (2015). "Cerebral toxoplasmosis in a patient on methotrexate and infliximab for rheumatoid arthritis." Intern Med 54(11): 1433-1436.
34. Radwan, A., et al. (2013). "Acute unilateral toxoplasma retinochoroiditis associated with adalimumab, a tumor necrosis factor-alpha antagonist." Retin Cases Brief Rep 7(2): 152-154.
35. Rao V, Schneider E, Proia AD, Fekrat S. (2014). Development of bilateral acquired toxoplasmic retinochoroiditis during erlotinib therapy. JAMA Ophthalmol. 132(9):1150-2.
36. Raquel CC, Lucía TC, Carmen VM, et al. (2020). Cerebral toxoplasmosis in patient with relapsing-remitting multiple sclerosis under treatment with alemtuzumab. Mult Scler Relat Disord. 39:101885.
37. Safa, G. and L. Darrieux (2013). "Cerebral toxoplasmosis after rituximab therapy." JAMA Intern Med 173(10): 924-926.
38. Savsek, L. and T. R. Opaskar (2016). "Cerebral toxoplasmosis in a diffuse large B cell lymphoma patient." Radiol Oncol 50(1): 87-93.
39. Steeples, L. R., et al. (2016). "Real-time PCR using the 529 bp repeat element for the diagnosis of atypical ocular toxoplasmosis." Br J Ophthalmol 100(2): 200-203.
40. Van Den Noortgate R, et al. (2023). Concurrent Ocular and Cerebral Toxoplasmosis in a Liver Transplant Patient Treated with Anti-CD40 Monoclonal Antibody. Case Rep Infect Dis. 2023:5565575.
41. Vigilante R, et al. (2025). Toxoplasma Gondii Replication During Belatacept Treatment in Kidney Transplantation: A Case Report and a Review of the Literature. Genes (Basel). 16(4):391.
42. Walkden, A., et al. (2020). "Atypical Ocular Toxoplasmosis During Adalimumab Anti-Tumor Necrosis Factor Therapy for Rheumatoid Arthritis." J Clin Rheumatol 26(8): e279-e280.
43. Young, J. D. and B. S. McGwire (2005). "Infliximab and reactivation of cerebral toxoplasmosis." N Engl J Med 353(14): 1530-1531; discussion 1530-1531.
44. Zecca C, Nessi F, Bernasconi E, Gobbi C. (2009). Ocular toxoplasmosis during natalizumab treatment. Neurology. 73(17):1418-9.

Table S5: Compilation of clinical vignettes of the 46 toxoplasmosis cases in patients on biologics or small molecules for autoimmune, oncologic or transplant conditions.

	Author, Year, PMID	Clinical Vignette	Toxoplasmosis manifestation	targeted immunotherapy	acute vs reactivation
1	Nardone et al. 2014 24191065	A 67-year-old female from Italy, with a past medical history significant for rheumatoid arthritis for 24 years, started on adalimumab due to failure of standard treatment presented with bilateral peripheral facial palsy and mild left hemiparesis. Brain MRI showed large round ring-enhancing lesions in the right thalamus. Brain biopsy showed perivascular lymphocytic infiltrates with areas of necrosis. T. gondii PCR was positive. No risk factors for toxoplasmosis were reported. Adalimumab was discontinued and patient was started on pyrimethamine, sulfadiazine and folinic acid, for 8 weeks. At that time the patient had residual bilateral facial palsy.	cerebral toxoplasmosis	adalimumab	unclear
2	de Almeida et al. 2022 36009113	A 62-year-old female from Portugal, with severe rheumatoid arthritis for 10 years in whom adalimumab was recently added 2 months prior to presentation to her multidrug RA regimen (prednisolone, leflunomide, methotrexate), presented with a 2-week history of worsening temporal disorientation and unsteady gait with grade 3 left hemiparesis. Also associated lymphopenia (ALC=600 µL). T. gondii serology was IgG positive, IgM negative. Brain CT showed a very large hypodense space occupying mass lesion with mass effect and surrounding edema, in the right basal ganglia. MRI showed the mass lesion was heterogeneous on T2/FLAIR with ring-enhancement with gadolinium; and on the PWI (perfusion weighted imaging) there was significant decrease of blood flow within the lesion. A brain Bx was performed; the brain smear showed necrosis and cystic structures containing encapsulated microorganisms, raising the hypothesis of toxoplasma infection and the final brain biopsy results confirmed the presence of bradyzoites, and free tachyzoites with positive T. gondii immunohistochemistry, establishing the diagnosis of cerebral toxoplasmosis. Patient was treated with P/S/FA (and steroids to decrease the brain edema). Adalimumab was discontinued. During the first 3 weeks patient showed mild clinical and radiologic improvement and lymphopenia resolved. Following de-escalation of prednisolone, the patient showed better clinical response with progressive motor improvement. At discharge patient had mild residual grade 4 hemiparesis. Completed 6 weeks course of P/S/FA. At 6 month follow up, patient was fully recovered from the motor deficit. The patient was subsequently treated for her RA with methotrexate, leflunomide, and prednisone and was placed on prophylactic TMP-SMX (160/800 mg PO BID) due to risk of Toxoplasma reactivation.	cerebral toxoplasmosis	adalimumab	reactivation
3	Cren et al. 2016 27478763	64 y woman from France with medical history of erosive rheumatoid arthritis for 1 year; was treated with adalimumab plus methotrexate plus prednisone for 2 months (after failure to reach adequate clinical response initially with methotrexate for 5months and subsequently to methotrexate plus etanercept for 3 months). After 2 months of this Rx while clinical response was satisfactory, patient developed one episode of tonic clonic seizures without other neurologic signs. Brain CT and Brain MRI showed multiple bilateral supratentorial singe-shaped brain lesions, contrast enhancing. CSF analysis was negative; CSF Toxo PCR was negative; Brain Biopsy showed plasma cells and lymphocytic infiltrates. Immunohistochemistry was positive for T. gondii and Brain Biopsy Toxo PCR was positive. Toxoplasma serology confirmed reactivation. High positive Toxo IgG, and high positive Toxo IgM and Toxo IgA. Comparison with Toxoplasma serology from 1 year earlier confirmed reactivation due to a high rise of Toxo IgG and IgM titers (from previously low positive Toxo IgG and negative IgM). Adalimumab and methotrexate were discontinued. Patient was treated with TMP/SMX (after the initial high dose of TMP/SMX: 320/1600 BID, patient developed AKI and the dose of TMP/SMX was decreased to 160/800 BID). Information about the clinical outcome, treatment duration, secondary prophylaxis and any changes in the immunotherapy after completion of the anti-Toxoplasma therapy were not reported.	cerebral toxoplasmosis	adalimumab	reactivation
4	Lim et al. 2007 16806484	63 Y male from the UK with history of chronic myelomonocytic leukemia, status post Alemtuzumab based HSCT (Fludarabine-Busulphan-Alemtuzumab). TMP/SMX prophylaxis was given as patient was Toxo R+/D- pre HSCT. 4 months post HSCT patient developed seizures and R sided weakness. Brain MRI showed left sided superior frontal gyral lesion with ring enhancement and edema. Patient was also receiving Cyclosporin A for grade II GVHD of the skin. Toxoplasma	cerebral toxoplasmosis	alemtuzumab	reactivation

		serology showed Toxo IgG+, IgM-. Although CSF Toxo PCR was negative, brain biopsy confirmed the Dx of Toxoplasmosis. Pyrimethamine/Sulfadiazine was started, and patient's condition was stabilized. Serial Brain MRI showed no increase in the size of lesions. (Although patient died 360 days post HSCT, this was from relapse of AML, not from toxoplasmosis)			
**5**	Lim et al. 2007 16806484	22 y male from South America, with AML, after induction chemotherapy with FLAG, received an Alemtuzumab based HSCT (TBI-busulphan-cyclophosphamide conditioning with Alemtuzumab). Toxo R+/D- HSCT. 2 months post HSCT, developed fever, confusion and memory loss. Patient was also on Cyclosporin prophylaxis for GVHD prophylaxis. His serum Toxo IgG and IgM were now positive. Brain CT showed subcortical changes in both frontal lobes with no enhancement. CSF analysis showed slightly elevated CSF protein. CSF Toxo PCR was negative. Patient was started on pyrimethamine/sulfadiazine (among other empiric therapies). Repeat MRI showed worsening of brain lesion, with involvement also of the temporal lobes. Due to marked clinical deterioration a brain biopsy was not performed. Patient died 93 days post HSCT (~1 month after the onset of symptoms). Postmortem brain biopsy confirmed the diagnosis of cerebral toxoplasmosis.	cerebral toxoplasmosis	alemtuzumab	reactivation
**6**	Raquel et al. 2020 31838310	Patient is a 43-year-old female from Spain, with history of relapsing remitting multiple sclerosis, was previously treated with interferon-B, corticosteroids, immunoglobulins, natalizumab, and fingolimod x 14 years. She did not receive DAMRDs for 1 year, suffered three severe outbreaks during the period, and she was then started on alemtuzumab. Two weeks after starting alemtuzumab treatment, she was admitted to the hospital for fever, headache, and somnolence. Brain MRI revealed multiple hyperintense nodular lesions with edema and nodular or ring uptake (in addition to the known demyelinating lesions). Given radiological characteristic findings and severe lymphopenia, toxoplasmosis was considered the most likely etiology and started empirical treatment with pyrimethamine and sulfadiazine (P/S). One day after starting P/S experienced a secondary generalized focal seizure that was treated symptomatically with good response. Serum Toxoplasma IgG and IgM antibodies were negative (in the context of severe lymphopenia). No CSF analysis was done due to the presence of mass effect from the brain lesions. At 1 month follow up, brain MRI showed marked improvement; lymphopenia resolved. The good clinical response to anti-Toxoplasma therapy further supported the diagnosis of cerebral toxoplasmosis. Patient was discharged home 40 days after admission and was placed on secondary prophylaxis with pyrimethamine and sulfadiazine for 1 year, until complete resolution of brain MRI lesions and CD4+ remained >200/µL for more than 6 months. She was not prescribed a second round of Alemtuzumab.	cerebral toxoplasmosis	alemtuzumab	unclear
**7**	Vigilante et al 2025 40282351	Patient is a 71-year-old male from Italy, kidney transplant recipient, on Belatacept therapy, after failing immunosuppression with tacrolimus based triple therapy (development of graft rejection and side effects); presented 8 months after initiation of Belatacept with left brachial and lower extremity weakness. CT and MRI of the brain showed large (5.3 cm x3.7 cm) nodular ring enhancing lesion/brain abscess in the right hemisphere. TMP-SMX was started empirically. CSF was positive for T. gondii, confirming diagnosis of cerebral toxoplasmosis due to reactivation. Of note patient pre-transplant was T. gondii IgG positive (D+/R+ status). TMP-SMX was continued for 6 weeks and Belatacept was suspended. During anti-Toxoplasma treatment, patient had progressive resolution of neurological symptoms and after 6 weeks of therapy the toxoplasmic brain abscess had significantly regressed.	cerebral toxoplasmosis	belatacept	reactivation
**8**	Kersten et al. 2019 31722870	A 71-year-old man from Netherlands, with relapsed/refractory diffuse large B-cell lymphoma (DLBCL) underwent first line Rituximab based immunotherapy (R-CHOP); due to refractory disease continued with second line immunotherapy (R-DHAP, R-VIM, R-DHAP), followed by high-dose chemotherapy with BEAM, and an autologous stem cell transplant. Few months later, his lymphoma relapsed, and the patient was received CAR T-cells (CTL019) after bridging chemotherapy and one cycle Rituximab-Bendamustine and lymphodepleting chemotherapy. On day 1 developed fever, that resolved after 5 ds and considered to be grade 1 cytokine release syndrome (CRS); no tocilizumab or steroids were warranted. On day 7 post-CAR T cell infusion, patient noted to have neurocognitive deficits. First noted to have deficits in the routine post-CAR T-cell therapy mini-mental testing scores and micrographia. Patient also reported flu like symptoms, numbness in legs, balance problems and hand tremor. Initially suspected to be ICANS, but brain MRI findings were suggestive of cerebral toxoplasmosis. Brain MRI showed hypointense lesion	cerebral toxoplasmosis	CAR T-cell	unclear

		with incomplete ring enhancement in the left basal ganglia with eccentric target sign surrounded by hypointense perifocal edema. Toxoplasma serology was negative, but likely due to profound lymphopenia due to prior immunosuppressive treatments. He started Rx with pyrimethamine, sulfadiazine, F/A and his neurologic symptoms improved within a few days and resolved fully. The patient remained in complete remission 21 months post–CAR T-cell therapy, however, his family reports mild residual cognitive impairment, with memory gaps and emotional lability (feeling of urgency to do things without delay, getting upset easily).			
**9**	Marzolini et al. 2019 31250826	46-year-old patient, from UK, with history of relapsed B-cell ALL following allogeneic sibling HSCT who was recently treated with CD19 chimeric antigen receptor (CAR) T-cell therapy 4 months prior; presented with weakness, imbalance, cognitive impairment and seizures. Brain CT revealed extensive white matter abnormality in bilateral frontal lobes and corpus callosum, and brain MRI showed large peripherally enhancing lesion in the right frontal lobe, extending across the corpus callosum into the left frontal lobe. Brain biopsy showed dense lymphocytic infiltrates (CD3+CD20-) and CD68 macrophage infiltrates; immunohistochemistry was positive for T. gondii tachyzoites; and CSF T. gondii PCR. was positive. (Patient was on azithromycin prophylaxis for Toxo R+/D- status after the preceding HSCT) Treatment was started with oral pyrimethamine, sulfadiazine, and folinic acid x6 weeks and continued with maintenance anti-Toxoplasma therapy with pyrimethamine (25 mg/day), sulfadiazine (500 mg QID). At 1 year follow-up, patient had near complete neurologic recovery with residual proximal muscle weakness of the right arm. Repeat brain MRI showed improvement of the Right frontal lobe lesions, whereas the corpus callosum lesions remained the same. Repeat brain biopsy showed no evidence of active infection or other pathology.	cerebral toxoplasmosis	CAR-T-cell	reactivation
**10**	Enriquez-Marulanda et al. 2017 29141790	30 y female from Colombia, with a 4-year history of multiple sclerosis (MS) who was treated for the last 1 year with fingolimod, presented with 1 week history of left sided hemiparesis, fever and fatigue. Brain MRI showed multiple demyelinating like lesions in the corticomedullary junction that could have corresponded to tumefactive form of MS relapse. Patient initially received methylprednisolone, for presume tumefactive MS acute relapse, but there was worsening of the hemiparesis, generalized malaise and fevers. Patient had leukopenia (WBC=2700 cells/mm3; lymphopenia=200 cells/mm3; and CD4=200 cells/mm3). CSF analysis showed lymphocytic pleocytosis (CSF WBC=36, 100% lymphocytes), protein 36 mg/dl). CSF Toxo PCR was positive. Toxo IgG and IgM were also positive. Patient was treated with Pyrimethamine plus IV clindamycin (due to possible TMP/SMX allergy). Total duration of Rx was not reported (patient continued Rx for 2 weeks after discharge). Symptomatic improvement was noted after day 5 of Rx. At 2 months follow up the Brain MRI showed significant improvement of brain lesions, especially those with contrast ring enhancement. At one year follow up, the patient has remained stable without other complications of the treatment. Fingolimod was suspended and patient was treated again with interferon beta 1A. // The authors pointed that differentiation between tumefactive MS and cerebral toxoplasmosis pauses a diagnostic challenge//	cerebral toxoplasmosis	fingolimod	acute
**11**	Misra et al. 2016 28011850	Patient is a 25-year-old male from India, with a history of sarcoidosis, who was recently changed to golimumab for 1 month presented with a frontal and left-sided headache after his 2nd monthly infusion. Work up revealed multiple irregular heterogeneous ring enhancing lesions with hemorrhagic foci and brain biopsy with T. gondii immunohistochemistry revealed trophozoites of T. gondii with perivascular histiocytes, consistent with cerebral toxoplasmosis. Treatment was initiated with TMP-SMX. Patient's clinical course was complicated by seizures refractory to anti-epileptics and the patient expired subsequent to a status epilepticus episode from irreversible anoxic brain injury.	cerebral toxoplasmosis	golimumab	unclear
**12**	Pulivarthi et al. 2015 26028002	Patient is a 76-year-old female from the US, with a history of rheumatoid arthritis, on infliximab and methotrexate, who presented with facial droop, slurred speech and difficulty walking, right sided weakness, memory loss and aphasia. Workup with head CT revealed extensive vasogenic edema involving the left cerebrum and a large 2.9 cm irregular lesion in the left putamen with ill-defined decreased attenuation with mass effect. Serology was T. gondii IgG positive and IgM negative and Right thalamic brain biopsy showed extensive necrosis, inflammation with lymphocytes and plasma cells consistent with abscess and encysted organisms consistent with trophozoites, indicating cerebral toxoplasmosis. Patient responded well to treatment with pyrimethamine, clindamycin, and leucovorin (as the patient	cerebral toxoplasmosis	infliximab	reactivation

		had history of sulfa allergy) and was discharged to transitional care unit after remaining neurologically stable.			
**13**	Young et al. 2005 16207863	Patient is a 36-year-old female from US, with history of rheumatoid arthritis, on infliximab, prednisone, methotrexate, and leflunomide; with the dose of infliximab being gradually increased until control of symptoms of RA, presented with diffuse headache, slurred speech, focal weakness, and a grand mal seizure. On exam, the patient had a left sided facial droop and hemiparesis in the left upper extremity. Workup revealed two lesions in right hemisphere on brain MRI, brain biopsy with T. gondii bradyzoites and tachyzoites, and serology for T. gondii was IgG positive and IgM negative. Infliximab was considered responsible for the reactivation, given the temporal proximity between the increase in the infliximab dose and the reactivation of toxoplasmosis. Treatment was initiated with pyrimethamine, folinic acid, and dapsone (dapsone used instead of sulfadiazine, because of a known sulfa allergy). Patient quickly had a substantial neurologic improvement.	cerebral toxoplasmosis	infliximab	reactivation
**14**	Hill et al. 2020 32670684	Patient is a 70-year-old female from the US, with history of rheumatoid arthritis on chronic therapy with methotrexate and infliximab, who presented with a 2 week history of right sided weakness with minor falls. Workup was significant for bilateral large ring-enhancing lesions at the basal ganglia (4x2 cm on the Left and 1 cm on the right) and bilateral cerebral edema. Brain biopsy done due to suspicion for brain metastases showed necrotic brain tissues and tachyzoites, consistent with cerebral toxoplasmosis. Immunohistochemistry for Toxoplasma was positive, and patient had high Toxo IgG titers. (Results for Toxo IgM were not reported). Dual immunosuppressive therapy was discontinued, and patient was treated with high-dose TMP-SMX for 6 weeks. Patient also received dexamethasone due to the cerebral edema. Patient had a complication post biopsy, with post-biopsy brain hemorrhage, and unfortunately the patient required to be transferred to acute-care hospital for continued monitoring.	cerebral toxoplasmosis	infliximab	unclear
**15**	Martin et al. 2006 16758413	Among 9 reported US patients who received an Alemtuzumab based HSCT, there was 1 case of cerebral toxoplasmosis; that developed 169 days after the first dose of Alemtuzumab and despite TMP/SMX prophylaxis.	cerebral toxoplasmosis	not reported	reactivation
**16**	Savsek et al. 2016 27069454	Patient is a 62 year old female from Slovenia, with a history of systemic diffuse large B cell lymphoma, on rituximab, cyclophosphamide, vincristine, doxorubicin and prednisolone (R-CHOP) chemotherapy, presented , after the 8th cycle of R-CHOP with high fever, headache, and altered mental status, confusion, apathetic, with dysexecutive syndrome, symmetrical, ataxia, right upper extremity weakness, homonymous hemianopsia, photophobia and pancytopenia. Workup with brain MRI revealed multiple hyperintense parenchymal lesions with mild surrounding edema located in both cerebral and cerebellar hemispheres, the largest one located in the R occipital lobe, with moderate gadolinium enhancement and T. gondii PCR of CSF was positive and serology revealed borderline Toxo IgG positive and negative IgM, consistent with cerebral toxoplasmosis. Had CSF pleocytosis (27 WBC, mainly lymphocytes) and high CSF protein. A 6-week intensive anti-Toxoplasma treatment was initiated with sulfadiazine, pyrimethamine and folic acid (P/S/FA). Patient slowly began improving a few days after starting P/S/FA and became more alert. First f/up MRI at 6 weeks showed reduction of edema and lesion size with hemorrhagic inclusions in some of the lesions. 3 months after presentation the patient had only mild residual cognitive deficits. Second f/up MRI 5 months after presentation showed further reduction of brain lesions. Patient began secondary prophylaxis for toxoplasmosis, after completion of the primary treatment course, due to the need for maintenance therapy with rituximab for lymphoma remission.	cerebral toxoplasmosis	rituximab	reactivation
**17**	Castaño-Amores et al. 2021 32592604	A 22-year female from Spain, with history of dermatomyositis, on rituximab and azathioprine for ~ 4 years, presented (9 months after rituximab (x11 cycles) and while also on azathioprine), with dysarthria and dysphagia, central facial paralysis and damage to the 9th and 10th cranial nerves, retrobulbar headache, vomiting and hiccupping. Head CT showed hypodense white matter lesions. Head MRI showed multiple supratemporal lesions in both hemispheres. T. gondii PCR of CSF was positive and patient started on pyrimethamine, sulfadiazine, and folinic acid. Symptoms improved and the patient was discharged. 15 days later, the patient was re-hospitalized with a manic episode (aggressiveness, nervousness, dysarthria, disorientation). Repeat head CT showed reduction in hypodense lesions in the thalamic and parietooccipital areas. Patient was diagnosed with psychosis 2/2 to cerebral toxoplasmosis and was treated with olanzapine and clonazepam. (of note, 10 days later, patient developed non obstructive nephropathy and AKI due to	cerebral toxoplasmosis	rituximab	unclear

		sulfadiazine induced crystalluria, which improved after sulfadiazine dose adjustment and alkaline fluid therapy). Due to sulfadiazine-related crystalluria, sulfadiazine was replaced by clindamycin 600 mg/6 h plus pyrimethamine 50 mg daily			
**18**	Safa et al. 2013 23589123	Patient is a 71-year-old female from France, with past medical history of treatment refractory cutaneous necrotizing vasculitis secondary to type 1 essential cryoglobulinemia, which resolved after completing 4 weekly infusions of rituximab, presented 4 months after completing rituximab tx with speech disturbance, behavioral changes and weight loss. Workup revealed multiple ring-enhancing lesions on brain MRI, T. gondii PCR of CSF was positive and serology for T gondii was IgG positive and IgM negative, consistent with cerebral toxoplasmosis. Although the patient was also on prednisone long prior to presentation, the patient developed toxoplasmosis only after the addition of rituximab. Treatment was initiated with pyrimethamine, sulfadiazine sodium, and folinic acid. However, despite treatments, patient's mental status did not improve.	cerebral toxoplasmosis	rituximab	reactivation
**19**	Lee et al. 2019 31909136	Patient is a 52-year-old female from the US, with a history of pemphigus vulgaris, on rituximab (after failure to respond to azathioprine, mycophenolate and prednisone). 6 weeks after the 3d cycle of Rituximab, the patient presented with persistent rhinorrhea (that was d/t CSF leak) and symptoms concerning for pseudotumor cerebri. Workup revealed encephalocele, CSF leak, and underwent repair with a lumbar drain. /// Brain MRI T2 weighted images revealed an ill-defined, round, T2 signal intense lesion in the right globus pallidus and lentiform nuclei with ring-like enhancement centrally. Toxo IgM was highly positive (35.9; with positive range>7.9), while Toxo IgG was negative. CSF Toxo PCR was negative, but the plasma next-generation cell free DNA sequencing assay (Karius test) was positive for T. gondii, consistent with acute cerebral toxoplasmosis. Treatment was initiated with pyrimethamine and leucovorin for 6 weeks, followed by trimethoprim-sulfamethoxazole (TMP-SMX), 3 times daily for another 6 weeks, then TMP-SMX, once daily as maintenance therapy. Mycophenolate was tapered to discontinuation and additional Rituximab Tx was deferred. After 2 weeks of pyrimethamine/leucovorin, there was complete resolution of abnormal signal at the R basal ganglia.	cerebral toxoplasmosis	rituximab	acute
**20**	Desmond et al. 2010 20029990	Patient is a 63-year-old male from Ireland, with a history of chronic lymphocytic leukemia, on rituximab (for 8 months prior to onset) along with fludarabine, and cyclophosphamide, who presented with apraxia, memory loss and cognitive impairment. Workup revealed high signal white matter on MRI, brain biopsy with ruptured toxoplasmosis cysts. (Of note, immunohistochemistry was positive for JC virus). CSF PCR was positive for both T. gondii and JC virus. Patient developed progressive ataxia, dysphagia, dementia and died 4 weeks after initial presentation. No report was made about anti-Toxoplasma treatment. Authors considered this case as a case of cerebral toxoplasmosis and progressive multifocal leukoencephalopathy.	cerebral toxoplasmosis	rituximab	unclear
**21**	Gonzalez-Vincent et al. 2019 30207078	Patient is a 17-year-old female from Spain, with a past medical history of Hodgkin lymphoma. She recently underwent allogeneic HSCT, initially discharged with cyclosporine and cotrimoxazole but later (Day +55 post HSCT) developed steroid refractory intestinal GvHD and was started on ruxolitinib as compassionate use. 3 weeks after starting ruxolitinib, she developed somnolence, altered mental status, and temporospatial disorientation. Workup revealed supra and infratentorial cortico-subcortical lesions, many with ring enhancement, PCR was positive for T. gondii in blood and CSF, consistent with acute cerebral toxoplasmosis. Patient also had severe lymphopenia, decreased NK cells and Regulatory T cells (Tregs). Ruxolitinib dose was decreased, and treatment was initiated with pyrimethamine-sulfadiazine-folinic acid and clindamycin. Subsequently, Toxoplasma seroconversion was observed alongside with improvement in consciousness. 7 days later, her consciousness level improved, but developed speech and swallowing problems, right vision loss, incontinence, symmetric weakness, hypotonia and areflexia. // Brain MRI lesions had improved and blood and CSF T. gondii PCR were negative. EMG showed axonal damage and VEP showed axonal and demyelinating lesions of the right optic nerve. Treatment for GBS was started; and she improved with recovery of speech, normalization of swallowing, increased visual acuity, control of the anal sphincter, and walking with help.	cerebral toxoplasmosis	ruxolitinib	acute
**22**	Hoellinger et al. 2021 33871919	Patient is a 38-year-old male from France, status post haplo-identical allograft for acute myeloid leukemia, who was started on cyclophosphamide, ciclosporin, mycophenolate-mofetil for post-transplant GvHD prevention. The patient presented	cerebral toxoplasmosis	ruxolitinib	reactivation

		3 weeks after transplant with acute diarrhea. Rectal biopsy showed grade II GvHD. Due to failure to respond to steroids, was changed to Tacrolimus, plus steroids, however, patient continued to have severe exudative enteropathy and abdominal CT showed diffuse enteritis and colitis. For concern for presumed GvHD the immunosuppressive Rx was intensified, and changed to Ruxolitinib, Tacrolimus, steroids. Enteric video capsule revealed diffuse ulcerations. New rectal biopsy showed no signs of acute GvHD //At the same time Blood PCR was positive for Toxoplasma; while Brain MRI, eye exam and chest CT showed no signs of toxoplasmosis dissemination at that time. Patient was started on IV clindamycin x 4 weeks, and clindamycin was discontinued after 3 consecutive negative blood PCR. // 1 month later (while on Rituximab, Tacrolimus and steroids), patient presented with left hemiplegia with brain CT showing capsulothalamic hemorrhage and MRI showing multiple nodular lesions on the gray matter; and positive CSF PCR for toxoplasma, consistent with cerebral toxoplasmosis. Of note, pre transplant Toxoplasma D+/R+ status. Empiric IV TMP-SMX was initiated for cerebral toxoplasmosis and ruxolitinib and tacrolimus were discontinued, while the steroid dose was decreased. Intestinal transit and neurological deficits improved 10 days after initiation of TMP-SMX. TMP-SMX treatment was continued for 12 weeks and after that patient was placed on PO TMP-SMX for secondary prophylaxis. At 6-month follow-up, the patient had full recovery of both neurological and digestive symptoms. (Of note, duodenal and jejunal biopsy Toxoplasma PCR were positive-at the time that the blood T. gondii PCR had become negative; suggesting intestinal toxoplasmosis. The GI symptoms of the presumed intestinal toxoplasmosis developed prior to starting the Ruxolitinib. However, the neurologic symptoms did not present until later, 1 month after being on Ruxolitinib, Tacrolimus and steroids.			
**23**	Bach et al. 2020 31965234	Patient is a 78-year-old female from Germany, with history of rheumatoid arthritis, on a TNF-a inhibitor and methotrexate, who presented with increasing weakness of bilateral lower extremities, significant gait and balance disturbances and dysdiadochokinesia (also restlessness, tremors, feeling weak with chills, headache and nausea). Serology was T. gondii positive for IgG (4,827 IU/ml) and negative for IgM, with high IgG avidity. (Patient also had +EBV WB for IgG and IgA and +PCR with low level EBV viremia). Brain MRI showed B/L space occupying lesions; diffusion weighted images showed centrally hyperintense lesions with surrounding hypointense rim; FLAIR images showed pronounced perifocal edema with ring enhancement. Brain biopsy revealed focal tachyzoites, consistent with cerebral toxoplasmosis. Treatment was initiated with sulfadiazine, pyrimethamine, and leucovorin but discontinued due to poor tolerance. Sulfadiazine was replaced with clindamycin due to development of erythroderma. Clindamycin was also subsequently replaced with atovaquone due to continued side effects. Treatment was continued for 10 weeks and prophylactic low dose atovaquone was initiated. Patient was stable at follow-up.	cerebral toxoplasmosis	TNF-a inhibitor	reactivation
**24**	Mejía-Salgado et al. 2024 38889439	A 70-year-old female from Colombia, with a past medical history significant for rheumatoid arthritis diagnosed at age 40, on Abatacept, corticosteroids and methotrexate, presented with complaints of bilateral progressively decreasing visual acuity and floaters over 3 months. Physical exam showed B/L granulomatous panuveitis, with diffuse chorioretinitis, associated with retinal necrosis with macular involvement. There were 0.5+ cells in the anterior chamber, posterior synechiae, nuclear lens sclerosis, central macular fibrosis, extensive retinal necrosis with fibrosis and +2 vitreous cells and haze. OD: hand movement only, OS: 20/80. Underwent vitrectomy. Abatacept and methotrexate were discontinued, and treatment was initiated with low-dose systemic corticosteroids and acyclovir and intravitreal ganciclovir, despite negative PCR for HSV 1-2 and VZV. 2 weeks after the vitrectomy, VA continued to deteriorate (VA OD: light perception only and OS: 20/200) with necrosis affecting the macula. Given no clinical improvement, the previous vitreous sample was analyzed further. Serology was IgG positive and IgM negative for Toxoplasma and PCR of vitreous fluid revealed presence of EBV and T. gondii. Treatment with oral clindamycin and TMP-SMX was initiated, and the patient also received 3 injections of intravitreal clindamycin, along with topical corticosteroids and cycloplegics. At 6-week follow-up, VA OD: counting fingers only with severe retinal necrosis with macular involvement; while in the OS there was improvement of the VA (OS: 20/70), with improvement of the macular edema, but residual retinal necrosis. Patient was started on TMP/SMX prophylaxis.	ocular toxoplasmosis	abatacept	reactivation
**25**	Biancardi et al. 2020	Patient is a 38-year female from Brazil, with a past medical history of Crohn's Disease, on adalimumab and azathioprine for at least 4 years, who presented with	ocular toxoplasmosis	adalimumab	acute

	32415301	blurred vision in the left eye during routine checkup. Workup was significant for yellow-white retinitis with blurred edges. Adalimumab and Azathioprine were discontinued and empiric TMP-SMX was initiated. Toxoplasma serology was positive for IgG and IgM. Due to reactivation of Crohn's Disease, azathioprine was reintroduced 23 days after ceasing immunotherapy and anti-Toxoplasma treatment was changed from TMP-SMX to sulfadiazine, pyrimethamine, and folinic acid (as best first line therapy). At follow-up, the patient had slow progressive improvement of ocular inflammation.			
**26**	Patnaik et al. 2024 37818617	A 21-year-old male from India with past medical history significant for juvenile idiopathic arthritis, on adalimumab and methotrexate for the past 4 years, presented with blurry vision. The patient's exam was significant for severe vitritis and patches of retinochoroiditis in the inferior aspect of the fundus, and no evidence of old chorioretinal scar. Serology for T. gondii was negative for IgG but positive for IgM and PCR of the aqueous aspirate was positive for T. gondii. These findings were consistent with ocular toxoplasmosis and patient's methotrexate, and adalimumab were discontinued and 8-week treatment commenced on co-trimoxazole, clindamycin, and tapering doses of oral corticosteroid. At 1 month follow up, patient's best corrected visual acuity had improved, best corrected VA OS was 6/9 (from 6/24 at presentation) with evidence of regressed chorioretinal lesion and significant reduction in vitritis. 3 months later, after being off adalimumab and methotrexate had a JIA flare up with anterior uveitis and joint pain and immunotherapy was re-initiated with topical corticosteroid and oral tofacitinib. Patient did not have recurrence of the toxoplasmic chorioretinitis at 6 months follow up	ocular toxoplasmosis	adalimumab	acute
**27**	Radwan et al. 25390810	Patient is a 47-year-old female from the US, with a history of ulcerative colitis on adalimumab and prednisone, who presented with sudden vision loss (VA OS=20/400). Work up was significant for vitritis and retinochoroiditis, with no old scar and T. gondii serology was positive for IgM and IgG, consistent with ocular toxoplasmosis. Adalimumab was withheld and treatment was initiated with sulfadiazine, pyrimethamine, folinic acid x 5 weeks, in addition to prednisone and x1 intravitreal injection of clindamycin. At follow-up, 2 months later, the patient had improved visual acuity (VA OS=20/30) and decreased size of plaques and infiltrates. OCT showed foveal atrophy with epiretinal membrane formations.	ocular toxoplasmosis	adalimumab	acute
**28**	Steeples et al. 2016 26174811	A 70-year-old female from the US, with past medical history of Crohn's Disease, on adalimumab and azathioprine, presented with floaters, reduced vision and inferior scotoma in her Right eye. Workup was significant for posterior uveitis, with anterior chamber cells, vitreous veils and a large area of active retinitis threatening the fovea and the optic nerve head, with widespread peripheral vasculitis; and was started on azithromycin. The T. gondii PCR of aqueous humor, came back positive, consistent with diagnosis of ocular toxoplasmosis. Immunosuppression therapy was temporarily discontinued, and treatment was initiated with 4 weeks of cotrimoxazole and clindamycin and oral prednisolone for 2 weeks. After 4 weeks, the uveitis had settled, and VA improved to 6/19. However, while tapering corticosteroids, there was recurrence of uveitis with vitritis and a new focus of retinitis adjacent to the previous lesion, with decrease in the VA to 6/38. A further course of clindamycin with atovaquone was initiated, with enhanced steroids to achieve full resolution of retinitis and uveitis. Patient remains off immunosuppression without flare up of Crohn's disease and VA 6/24.	ocular toxoplasmosis	adalimumab	reactivation
**29**	Kayabaşı et al. 2024 38655271	A 52-year-old female from Turkey, with past medical history significant for ankylosing spondylitis, on adalimumab, salazopyrin, and prednisolone, presented with chief complaint of left-sided decreased vision x 4 months. VA was hand motion only on the left eye. Patient's exam was significant for chorioretinitis, and serology revealed IgG positive and IgM negative for T. gondii, consistent with ocular toxoplasmosis. Adalimumab was discontinued and patient was treated with TMP-SMX 180/600 BID, azithromycin 500 mg daily, and x2 intravitreal injections of clindamycin due to persistent vitritis, and received also topical prednisolone acetate 1% eye drops, and tropicamide 0.5% eye drops. At 5 months follow up, patient's left VA had improved to 4/10, s-lamp examination was unremarkable, and intraocular pressure was 13 mmHg, vitreous haze lessened, and lesion appeared atrophic with sharp borders. Patient received 5 months of TMP/SMX and 2 months of azithromycin.	ocular toxoplasmosis	adalimumab	reactivation
**30**	Walkden et al. 2020 31295158	A 86-year-old female from UK, with past medical history of rheumatoid arthritis, on adalimumab monotherapy, presented with floaters and reduced vision in her right eye. Patients had significant vitritis and a large area of necrotizing retinitis. Empiric	ocular toxoplasmosis	adalimumab	acute

		cotrimoxazole was initiated. PCR of aqueous humor was positive for T. gondii, and serology revealed positive IgM and negative IgG. Adalimumab was discontinued and clindamycin was added to TMP/SMX for 4 weeks, plus oral prednisolone. At 3 months, the uveitis was inactive with transformation to a retinochoroidal scar; VA was 6/15. Adalimumab was not restarted. Unfortunately, at 6 months patient underwent an elective right vitrectomy to remove the post inflammatory debris (along with cataract surgery) but then developed severe recurrence of retinitis with significant and permanent vision loss in the right eye, to hand movement only.			
**31**	Lanfranco et al. 2016 27614031	A 74-year-old male, from France, with past medical history of kidney allograft transplant for anti-PR3 ANCA vasculitis, on belatacept, mycophenolic acid, and prednisone, presented with a complaint of acute right eye blindness 11 years after transplantation. Work up revealed chorioretinitis and ischemic vasculitis and aqueous humor T. gondii PCR and T. gondii serology were both positive, consistent with ocular toxoplasmosis. Treatment was initiated with pyrimethamine and sulfadiazine for 6 weeks. Unfortunately, at 3-month follow-up, no recovery was observed. There was no report on whether the biologic agents/and/or the other immunosuppressive medications were modified. (Of note patient was Toxoplasma D+/R- prior to transplant but developed severe ocular toxoplasmosis 11 years after transplant after the consumption of undercooked meat. After the episode of ocular toxoplasmosis, the patient developed also positive Toxoplasma serology).	ocular toxoplasmosis	belatacept	acute
**32**	Rao et al 2014 24993473	Woman in her early 60's, from the U.S., undergoing treatment for metastatic lung adenocarcinoma with erlotinib, presented with bilateral floaters and decrease vision on the right eye. Patient was regularly consuming wild venison. 3 months prior to presentation patient (and her husband) consumed consumption of undercooked wild venison, and at that time patient (and her husband) developed prolonged fever and flu-like illness. On exam, patient had 2+ vitritis bilaterally and 3 peripapillary patches of retinitis in the right eye and a single focus of retinitis in the perifoveal region of the left eye. There were no old retinal scars detected. Serology showed strong positive T. gondii IgM, IgG, IgA, and IgE titers, suggestive of recent acute Toxoplasma infection with bilateral ocular disease (of note her husband also had serologic evidence of recent acute Toxoplasma infection). She was treated with oral atovaquone and at follow up, vitritis had cleared and vision had returned to baseline. Patient expired 2 months after initiating atovaquone therapy; no details for the cause of death were reported in the paper (but likely from her underlying disease. Post mortem exam showed full-thickness retinal necrosis and a T. gondii cyst in the area of previously clinically detected retinitis; but there was no post-mortem report for toxoplasmosis in other organ tissues reported in the paper).	ocular toxoplasmosis	erlotinib	acute
**33**	Lassoued et al. 2007 17067660	Patient is a 40-year-old female from France, with a history of rheumatoid arthritis, recently started on etanercept (in addition to methotrexate and prednisone) and 4 weeks later presented with vision loss in the lower quadrant of her left eye. Workup was significant for chorioretinitis (with peripapillary edema and intense vitreal inflammation and choroidal occlusion at the fluorescent angiography) with positive IgG and negative IgM for T. gondii, consistent with ocular toxoplasmosis d/t reactivation of toxoplasmosis. Treatment with pyrimethamine, sulfadiazine, and folinic acid was started. Patient was noted to have moderate improvement with a retinal scar suggestive of sequelae of toxoplasmic chorioretinitis.	ocular toxoplasmosis	etanercept	reactivation
**34**	Basu et al. 2010 20128654	48 y male from India, with medical history of chronic myeloid leukemia (CML) , on imatinib for 4 years, with CML in remission, developed gradual painless decrease in vision in both eyes, with visual equity at counting fingers only at 2 meters. Had mild vitritis with full thickness chorioretinal patches, intraretinal hemorrhages and retinal detachment. There was no old chorioretinal scar. Vitreous fluid T. gondii PCR was positive and Toxoplasma serology showed very high Toxo IgG titers, but negative Toxo IgM. Was treated with TMP/SMX (160/800) BID for 6 weeks and oral prednisone with tapering over 5 weeks. Imatinib was continued. At 3 months f/up the chorioretinitis had completed resolved and the VA had improved (OS 20/30 and OD 20/40). This bilateral ocular toxoplasmosis was considered to represent toxoplasmosis reactivation and disease resolved without discontinuation of imatinib. There was no reference made about secondary prophylaxis.	ocular toxoplasmosis (B/L)	imatinib	unclear
**35**	Lassoued et al 2007 17067660	Patient is a 43-year-old male from France, with a history of rheumatoid arthritis, on infliximab and methotrexate, who presented with sudden decreased vision of the left eye, 3 months after the addition of the infliximab. Workup revealed chorioretinitis and PCR of aqueous fluid was positive for T. gondii, consistent with ocular toxoplasmosis. Also, serum T. gondii IgM was positive and aqueous humor T. gondii IgM was positive. Infliximab was discontinued and he was treated with	ocular toxoplasmosis	infliximab	acute

		pyrimethamine, sulfadiazine, and folinic acid for 6 weeks. At 18-month follow-up, the patient had central scotoma secondary to scarring due to toxoplasmic chorioretinitis.			
**36**	Zecca et al. 2009 19776379	Patient is a 28-year-old male from Switzerland, with relapsing remitting multiple sclerosis (RRMS), eventually treated with natalizumab (After 7 prior relapses); s/p 11 infusions of natalizumab over a 10-month period, who presented with floaters and decreased vision in the left eye. Physical exam revealed iritis and vitritis, and 1 acute peripapillary gray, white retinal necrosis with adjacent old retinal scars and serology showed high positive IgG titers and negative IgM for T. gondii, consistent with reactivated ocular toxoplasmosis. (Negative CSF T. gondii and negative brain MRI excluded CNS Toxo disease). Natalizumab was suspended and patient was treated with pyrimethamine, sulfadiazine, folinic acid (with prednisone taper) for 5 weeks with prompt reduction in ocular lesions, confirming the diagnosis of ocular toxoplasmosis reactivation.	ocular toxoplasmosis	natalizumab	reactivation
**37**	Goldberg et al. 2013 23944322	Patient is a 65-year-old male from the US, with a history of polycythemia vera, on ruxolitinib, who presented with decreased vision in both eyes. Workup revealed decreased visual acuity in both eyes x 1mo (VA=counting fingers only on the left eye), focal retinal whitening, and vitritis in bilateral eyes, with no adjacent chorioretinal scars. PCR of aqueous fluid was positive for T. gondii and serology revealed positive Toxoplasma IgM and IgG. Patient was weaned off ruxolitinib and started on trimethoprim–sulfamethoxazole and clindamycin. At 3 month follow up, the patient had stable vision with retinal scarring.	ocular toxoplasmosis	ruxolitinib	acute
**38**	Javadzadeh et al. 2020 32212270	Patient is a 60-year-old female from the UK, with a past medical history of moderate to severe psoriasis, on ustekinumab, presented with floaters in her right eye. Patient's workup was significant for chorioretinitis, and serology was positive for T. gondii, consistent with ocular toxoplasmosis. Treatment was initiated with 1 month of oral azithromycin, pyrimethamine, folinic acid, and topical dexamethasone with full resolution of inflammation after 1 month and development of a quiescent scar. The patient was continued on ustekinumab after the 1st occurrence. Four months later, the patient presented with complete vision loss of the right eye, consistent with recurrence of ocular toxoplasmosis involving the fovea. Patient was treated with the initial regiment with added oral prednisolone and topical dorzolamide and after the 2nd episode, the inflammation almost completely resolved after 1 week, but d/t involvement of the fovea, pt had permanent vision impairment. During the second episode, ustekinumab was temporarily withheld, but then restarted at a later time.	ocular toxoplasmosis	ustekinumab	acute
**39**	Kator et al. 2020 31958891	54 y male from the US, with relapsed/refractory diffuse large B cell lymphoma, received CAR-T therapy. His immediate post-CAR-T therapy course was complicated by grade 2 CRS and grade 4 neurotoxicity which were treated with three doses of tocilizumab and steroids, followed by an oral dexamethasone taper which he completed on day 24. Patient was neutropenic until day 26 post CAR-T and remained profoundly lymphopenic with an ALC=200/µl. Trimethoprim/sulfamethoxazole (TMP-SMX) prophylaxis was not initiated due to persistent cytopenia. On day 67 post-CAR-T, patient was readmitted with headache, progressive weakness, anorexia and intermittent fevers. Was given a dose of tocilizumab for possible CRS, with no improvement. Developed respiratory failure and required intubation. Chest CT showed diffuse dense ground-glass lung opacities. TMP-SMX was initiated for possible PJP infection. Patient developed haemophagocytic lymphohistiocytosis with ANC=1500/mcL; ALC=300/mcL; PLT=36,000/mcL; Ferritin>44,000 ng/ml; soluble IL2 9,630 pg/ml (nl <1033). Despite aggressive Tx patient's clinical status rapidly declined, patient developed multiorgan failure and died on day 70 after CAR-T therapy. Autopsy showed disseminated toxoplasmosis with Toxoplasma tachyzoites and tissue cysts in the CNS (cerebrum, cerebellum, medulla, cervical spine), and Lungs, with extensive lung involvement with neutrophilic infiltrates, hemorrhages and Toxoplasma cysts and debris. Toxoplasmosis was confirmed also by immunohistochemistry.	disseminated toxoplasmosis (including pneumonic toxoplasmosis)	CAR-T	reactivation
**40**	Van Den Noortgate et al. 2023 37545749	70-year-old male from Belgium, with history of liver transplant 1 year prior, treated with iscalimab to prevent rejection, presented with right upper extremity weakness, gait instability for 4 weeks (with right sided spastic paresis, hyperreflexia, hypertonia, ankle clonus). Also had decreased visual equity on the left eye, with a large necrotic retinal lesion of the left eye, 0.5+cells in anterior chamber and mild vitritis. MRI revealed a 1.5 cm contrast-enhancing lesion in the left basal ganglia with perilesional edema and slight mass effect. TMP-SMX 800/160 mg BID was empirically initiated. CSF analysis was normal and CSF T. gondii PCR was negative.	disseminated toxoplasmosis	iscalimab	reactivation

		PCR of the aqueous fluid came back positive for T. gondii and TMP/SMX BID was continued. Because of unchanged neurologic status in the weeks after presentation, a brain biopsy was performed which showed bradyzoites and immunohistochemistry was positive for T. gondii. (Of note 4 months prior to presentation patient was Toxo IgG was+, IgM-). TMP-SMX dose was increased to QID and treatment was continued for 6 weeks; iscalimab was also discontinued (switched to everolimus). Follow up brain MRIs also showed gradual decrease in the thalamic lesion and the surrounding edema and the retinitis lesions in both eyes gradually became atrophic. At 6 month follow up, there was marked improvement of neurologic status, with mild residual right sided hyperreflexia, ataxic gait; and the ocular findings were stable. TMP-SMX was continued as ongoing secondary prophylaxis (BID dosing)			
**41**	Gharamti et al. 2018 30460322	Patient is a 65-year-old male from the US, with a history of rheumatoid arthritis, on abatacept, and R orbit pseudolymphoma. Due to expansion of pseudolymphoma to the right orbit, the patient required radiation therapy. Received also 6 cycles of bendamustine and rituximab over 6 months. Due to progression of the disease on the MRI and rapid vision loss, started on trametinib. 5 weeks later presented with gait incoordination and difficulty in fine motor skills. Brain MRI showed multiple supra and infratentorial ring enhancing lesions, CSF lymphocytic pleocytosis, retinitis, myocarditis (with chest discomfort, dyspnea, orthopnea, elevated troponin and diffuse hypokinesis on ECHO), and right thigh myositis on MRI and leg pain. Serology confirmed acute Toxoplasma infection with positive Toxo IgG, IgM, IgA, and low IgG avidity at the PAMF-TSL lab. Patient reported recent consumption of wild boar sausages, prior to onset of the acute toxoplasmosis (while on trametinib). Presentation was consistent with acute disseminated toxoplasmosis. Treatment was initiated with a 16-week course of high dose TMP-SMX, resulting in substantial clinical improvement and reduction in size of CNS lesions at 16-week follow-up.	disseminated toxoplasmosis (including cerebral toxoplasmosis, myocarditis and myositis)	trametinib	acute
**42**	Azevedo et al. 2010 21253622	Patient is a 36-year-old male from Brazil, with a history of ankylosing spondylitis, on adalimumab for 23 months, who presented with cervical and axillary lymphadenomegaly, malaise, and headache. Workup revealed normal CT and ophthalmologic exams and T. gondii serology revealed initially positive IgM and negative IgG. Adalimumab was discontinued and anti-Toxo treatment was initiated with TMP-SMX and spiramycin. LADP improved within a week and after 2 months had complete resolution of symptoms. F/up serology showed seroconversion with slightly positive IgG and negative IgM. Adalimumab was reintroduced 3 months after complete resolution of symptoms and at 6 months follow up, patient had remained well.	lymphadenopathy	adalimumab	acute
**43**	Lobo et al. 2020 32149179	Patient is a 34-year-old female from Australia, with a history of psoriasis, on ixekizumab, who presented with a 3-month history of left axillary lymphadenopathy, dry cough, rhinorrhea. Workup showed positive Toxo IgG and IgM at high titers and low IgG avidity. Serial paired serology demonstrating increasing IgG titer, declining IgM, and persistence of low IgG avidity, consistent with acute primary Toxoplasma infection. Biologic was not discontinued, and treatment was given for only 14-days (pyrimethamine, leucovorin, and clindamycin). Patient was seen at the dermatology clinic every 3 months while continuing treatment with ixekizumab and up to 5 years posttreatment, patient has not recurrence of Toxoplasma infection.	lymphadenopathy	ixekizumab	acute
**44**	Muslimani et al 2019 31420435	Patient is a 26 year old male from Italy, with history of psoriasis vulgaris, on ustekinumab x 17 months, who presented with fatigue, intermittent low-grade fever, nocturnal sweating, generalized lymphadenomegaly and unexplained weight loss. Workup revealed lymphadenomegaly, splenomegaly and serology revealed high levels of Toxoplasma IgG and IgM and low IgG avidity. First stage of treatment was cessation of ustekinumab followed by close monitoring of symptoms and serology. At 3 month follow up, LADP had resolved and at 4 months f/up all patient's symptoms were resolved. Biologic was discontinued permanently. (Patient was HLA C*06 and HLA C*12, indicating enhanced effect of Ustekinumab)	lymphadenopathy	ustekinumab	acute
**45**	Martina et al 2011 20955469	39 y male from Spain with history of ESRD received and renal transplant and placed on a JAK-3 inhibitor monotherapy. 2 months after transplant, the JAK-3 inhibitor was discontinued due to anemia. Immunosuppression was changed to Tacrolimus, Mycophenolate. Patient 8 months post-transplant developed flu-like symptoms with headache and nonproductive cough. Patient was on TMP/SMX prophylaxis for PJP for 6 months after transplant. Subsequently developed also fever and moderate renal failure. Although condition was good, patient remained febrile. CXR showed slight bilateral interstitial infiltrates. Started on TMP/SMX high dose for possible PJP	pneumonic toxoplasmosis	JAK-3 inhibitor	acute

		but BAL was negative for PJP. Patient remained febrile for 4 weeks. Toxoplasmosis was then suspected due to Toxo Donor+/Recipient - status. Toxoplasma IgM was positive, while IgG was negative 1 month after admission. Seroconversion was documented with Toxoplasma IgM negative and Toxoplasma IgG positive 1 months after the initial diagnosis. Pyrimethamine/Sulfadiazine was started and continued for 6 weeks. CXR was normal 1 week after initiation of anti-Toxoplasma therapy. Subsequently placed on secondary prophylaxis with TMP/SMX (1 DS TIW) for 3 months. Patient remained asymptomatic 1 year post transplant. (Of note patient developed the pneumonic toxoplasmosis 2 months after discontinuing the 6mo PJP TMP/SMX prophylaxis)			
**46**	Krull et al 2021 34382299	Patient is a 36-year-old G2P1 female from Switzerland, with past medical history significant for ankylosing spondylitis, on adalimumab for 4 years, until 5 months before conception. Found to have abnormal fetal scan at 26 weeks gestation, with Right cerebral ventriculomegaly and multiple hyperechoic lesions in the cerebral parenchyma. Fetal MRI confirmed multiple subependymal T2 intense lesions and multiple cortical and subcortical cysts. Amniocentesis revealed fetal infection with positive amniotic fluid T. gondii PCR. Termination of pregnancy was requested by the patient. Fetal autopsy was consistent with disseminated severe toxoplasmosis, with the brain showing large necrotic and inflammatory lesions, calcifications in adrenal glands and hepatosplenomegaly. Retrospective serology evaluation revealed the patient had acquired the Toxo infection 6-7 months before conception when she was on adalimumab and the patient continued to have increasing T. gondii IgG titers for more than a year after the onset of the infection. Of note the mother was screened for toxoplasmosis at 6 weeks GA and was found to have + T.gondii IgG at very high titers, + IgM, but also had high IgG avidity, and thus it was considered at that time that the mother had a T.gondii infection at least 2 months prior to conception (and thus she was considered not at risk for vertical transmission; although high T. gondii IgG titers have been shown to be associated with high risk of vertical transmission). Moreover, the preceding fetal US at 20 weeks GA was normal; thus the vertical transmission occurred late in gestation but nevertheless led to a very symptomatic case of congenital toxoplasmosis.	congenital toxoplasmosis	adalimumab	acute
